# Quantitative transcription dynamic analysis reveals candidate genes and key regulators for ethanol tolerance in *Saccharomyces cerevisiae*

**DOI:** 10.1186/1471-2180-10-169

**Published:** 2010-06-10

**Authors:** Menggen Ma, Lewis Z Liu

**Affiliations:** 1 Bioenergy Research, National Center for Agricultural Utilization Research USDA-ARS, Peoria, IL USA; 2 Department of Computer Science, New Mexico State University, Las Cruces, NM USA

## Abstract

**Background:**

Derived from our lignocellulosic conversion inhibitor-tolerant yeast, we generated an ethanol-tolerant strain *Saccharomyces cerevisiae *NRRL Y-50316 by enforced evolutionary adaptation. Using a newly developed robust mRNA reference and a master equation unifying gene expression data analyses, we investigated comparative quantitative transcription dynamics of 175 genes selected from previous studies for an ethanol-tolerant yeast and its closely related parental strain.

**Results:**

A highly fitted master equation was established and applied for quantitative gene expression analyses using pathway-based qRT-PCR array assays. The ethanol-tolerant Y-50316 displayed significantly enriched background of mRNA abundance for at least 35 genes without ethanol challenge compared with its parental strain Y-50049. Under the ethanol challenge, the tolerant Y-50316 responded in consistent expressions over time for numerous genes belonging to groups of heat shock proteins, trehalose metabolism, glycolysis, pentose phosphate pathway, fatty acid metabolism, amino acid biosynthesis, pleiotropic drug resistance gene family and transcription factors. The parental strain showed repressed expressions for many genes and was unable to withstand the ethanol stress and establish a viable culture and fermentation. The distinct expression dynamics between the two strains and their close association with cell growth, viability and ethanol fermentation profiles distinguished the tolerance-response from the stress-response in yeast under the ethanol challenge. At least 82 genes were identified as candidate and key genes for ethanol-tolerance and subsequent fermentation under the stress. Among which, 36 genes were newly recognized by the present study. Most of the ethanol-tolerance candidate genes were found to share protein binding motifs of transcription factors Msn4p/Msn2p, Yap1p, Hsf1p and Pdr1p/Pdr3p.

**Conclusion:**

Enriched background of transcription abundance and enhanced expressions of ethanol-tolerance genes associated with heat shock proteins, trehalose-glycolysis-pentose phosphate pathways and PDR gene family are accountable for the tolerant yeast to withstand the ethanol stress, maintain active metabolisms, and complete ethanol fermentation under the ethanol stress. Transcription factor Msn4p appeared to be a key regulator of gene interactions for ethanol-tolerance in the tolerant yeast Y-50316.

## Background

Cellulosic ethanol production from renewable biomass including lignocellulosic materials and agricultural residues is a promising alternative to fossil oil as transportation energy [1-6]. Increased ethanol titer or concentration of microbial fermentation has been considered as a strategy to reduce energy cost in downstream distillation and waste treatment [7]. *Saccharomyces cerevisiae *is a traditional ethanol producer, yet it is sensitive to high concentrations of ethanol. Ethanol diffuses freely across biological membranes in yeast cells allowing equalization of ethanol concentrations between intracellular and extracellular pools. As a result, the increased ethanol concentration in a medium inhibits cell growth, damages cell viability, and reduces ethanol yield [8-10]. Using ethanol tolerant strains for high ethanol yield fermentation is desirable for cost-efficient ethanol production. However, mechanisms of ethanol tolerance are not well known and ethanol-tolerant yeast is not readily available.

More than 400 genes have been identified involving ethanol tolerance by high throughput assays [11-21]. Most genes are related to heat shock protein genes [11,21-23], trehalose biosynthesis and amino acid pathways [13,17,24,25], fatty acid and ergosterol [15,26-30]. While a significant amount of gene expression data was obtained over the past decade, a lack of solid characterization of expression dynamics exists. For example, studies using snapshot methods were common and often lower concentrations of ethanol were applied at late stages of cell growth (Table 1). When a serial of samples were taken over time, there is usually no additional ethanol challenge applied. As a result, it is very difficult to avoid biased assessment for the complex interactions of ethanol tolerance in yeast.

**Table 1:** Recent studies on gene expression response and genes related to ethanol tolerance for *Saccharomyces cerevisiae*

Method	Strain	Growth condition	Cell growth stage	Ethanol challenge concentration (%, v/v)	Sampling time-points	Reference
qRT-PCR Array	NRRL Y-50316	YM, 30°C	OD_600 _= 0.15	8	0, 1, 6, 24, 48 h	This work
	NRRL Y-50049					
Microarray	S288c	YPD, 28°C	OD_660 _= 0.8	7	0, 0.5 h	[11]
Microarray	PMY 1.1	YNB, 30°C	OD_620 _= 1.0	5	0, 1, 3 h	[12]
	FY834					
Microarray	S288c IFO2347	YPD, 30°C	OD_660 _= 1.0	5	0, 0.25, 0.5, 1, 2, 3	[13]
Microarray	FY834 A1	YPD, 30°C	Initial	10	log phase	[15]
Microarray	Vin13	Grape juice, 30°C	None	0	Varied ethanol concentrations	[16]
	K7					
	K11					
Microarray	K701 SR4-3	YPAD, 20°C	None	0	log phase	[17]
Microarray	EC1118	Synthetic must, 24°C	None	0	Fermentation stages1 to 6	[18]
	K-9					
Microarray	X2180-1A	YPD, 30°C	None	0	log phase	[19]
SAGE	EC1118	Synthetic must, 28°C	None	0	0, 20, 48, 96 h	[20]
Microarray	Kyokai no. 701	Sake mash, 15°C	None	0	2, 3, 4, 5, 6, 8, 11, 14, 17 day	[21]

Yeast tolerance to ethanol is complex involving multiple genes and multiple quantitative trait loci [31]. Development of ethanol-tolerant strains has been hindered by using conventional genetic engineering methods. On the other hand, yeast is adaptable to stress conditions under directed evolutionary engineering [2,32-34]. Adaptation and evolutionary engineering have been successfully applied in obtaining ethanol tolerant strains at varied levels [26,27,35,36]. Previously, we developed tolerant ethanologenic yeast *S. cerevisiae *NRRL Y-50049 that is able to withstand and *in situ *detoxify numerous fermentation inhibitors that are derived from lignocellulose-to-ethanol conversion such as furfural and 5-hydroxymethylfurfural (HMF) [33,37,38]. Building upon the inhibitor-tolerant yeast, we recently developed ethanol-tolerant yeast NRRL Y-50316 using an adaptation evolutionary engineering method under laboratory settings.

The qRT-PCR is an accurate assay platform and considered as an assay of choice for quantitative gene expression analysis. It is commonly used to confirm high throughput expression data obtained by microarray which has higher levels of variations from multiple sources. For absolute quantitative gene expression analysis, due to the necessary wells required for the construction of standard curves, very limited number of wells are available for target gene assays [37,39]. Recently, a significant advance has been made to safeguard data accuracy and reproducibility with two new components, a robust mRNA serving as PCR cycle threshold reference and a master equation of standard curves [37,40,41]. These developments allow unification of expression data from different experimental conditions for comparative analyses. This method and other similar approaches have been rigorously examined with demonstrated advantages of reliability and reproducibility over housekeeping genes [37,40-45].

In the present study, we compared cell growth, cell viability, ethanol production and gene expression under the ethanol stress between two very closely related strains, the lignocellulosic inhibitor-tolerant Y-50049 and its ethanol-tolerant derivative Y-50316 retaining the inhibitor-tolerance characteristics. Using the recently developed pathway-based qRT-PCR array assays, we investigated transcription dynamics of over 170 selected genes based on previous reports and our preliminary screening in response to ethanol challenge using a time-course study. The objective of this study was to identify candidate and key genes responsible for ethanol tolerance to support complete ethanol fermentation. Our results uncovered previously unreported genes accountable for ethanol tolerance and identified legitimate candidate genes of ethanol tolerance based on the ethanol-tolerant Y-50316. Results of this study will aid dissection of ethanol tolerance mechanisms in yeast and metabolic engineering efforts for more tolerant strain development.

## Results

### Tolerance and viability

**Figure 1:**
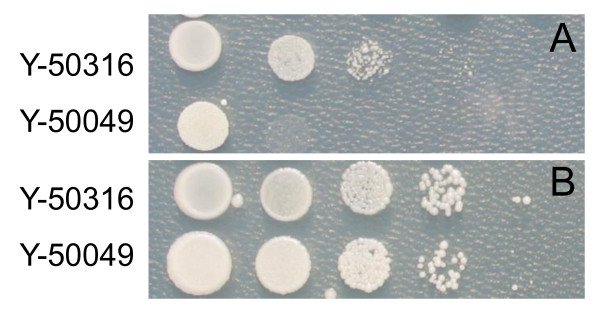
**Cell growth response to ethanol and inhibitors**. Comparison of cell growth and colony appearance for ethanol-tolerant and inhibitor-tolerant mutant *Saccharomyces cerevisiae *NRRL Y-50316 and its parental inhibitor-tolerant strain NRRL Y-50049 on YM plate of 2% glucose containing 8% (v/v) ethanol (A) or amended with inhibitors of furfural and 5-hydroxymethylfurfural each at 10 mM (B). The cultures initially applied were estimated with viable cell account of approximately 1.0 × 10^7 ^per ml as measured by colony forming units. A serial of 10-fold culture dilutions in water were spotted onto a medium plate containing ethanol or inhibitors and cell growth examined 7 days after incubation at 30°C.

On a solid medium of 2% glucose containing 8% ethanol, ethanol-tolerant strain Y-50316 showed cell growth from reduced cell concentrations at 10- to 100-fold dilutions (Figure 1A). In contrast, cells of Y-50049 failed to grow at any reduced cell concentration levels. Strain Y-50316, an ethanol-tolerant derivative from its parental Y-50049, maintained the inhibitor-tolerance and able to *in situ *detoxify furfural and HMF derived from pretreatment of lignocellulosic biomass. On a medium of 2% glucose containing furfural and HMF at 10 mM each, both strains showed similar growth patterns against the inhibitors at all cell dilution levels from 10- to 1000-fold (Figure 1B). On a liquid YM of 2% glucose containing furfural and HMF, both strains showed similar growth pattern and reached stationary phase in 30 h (data not shown)

**Figure 2:**
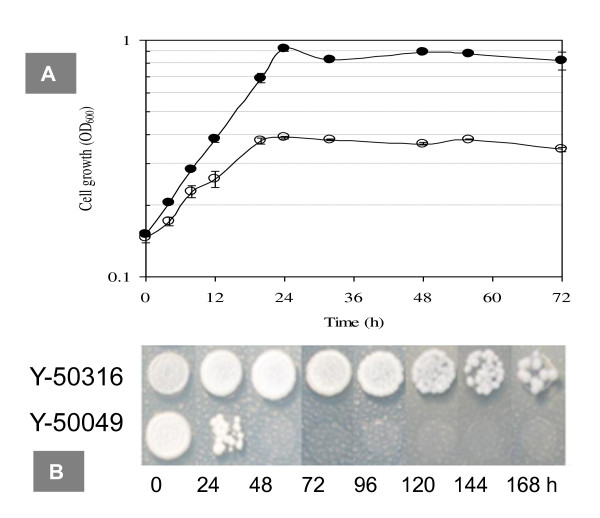
**Cell viability and growth under the ethanol stress**. Cell viability of ethanol- and inhibitor-tolerant mutant *Saccharomyces cerevisiae *NRRL Y-50316 (●) and its parental inhibitor-tolerant strain NRRL Y-50049 (○) in response to 8% (v/v) ethanol challenge as measured by OD_600 _on a liquid YM of 2% glucose (A) and culture appearance of cell growth on a solid YM of 2% glucose (B). The time point at the addition of ethanol to the medium was designated as 0 h. Cell growth on YM plate was evaluated 7 days after incubation at 30°C.

On a liquid YM of 10% glucose containing 8% ethanol, the ethanol-tolerant Y-50316 displayed a nearly normal growth and reached stationary phase 24 h after incubation (Figure 2A). For its parental strain Y-50049, cell mass was low and cell growth appeared ceased after 24 h. When cell viability was tested using solid YM of 2% glucose inoculated with the cell cultures at different time point, the parental strain Y-50049 showed a very poor growth response at 24 h and no viable cell growth was observed at any later time points (Figure 2B). On the other hand, the ethanol-tolerant strain Y-50316 displayed a normal growth for samples taken at 24 h till 96 h after the ethanol challenge. Reduced cell growth and cell lyses were observed for samples taken at 120 to 168 h after ethanol challenge when the fermentation was completed for several days.

### Glucose consumption and ethanol production

**Figure 3:**
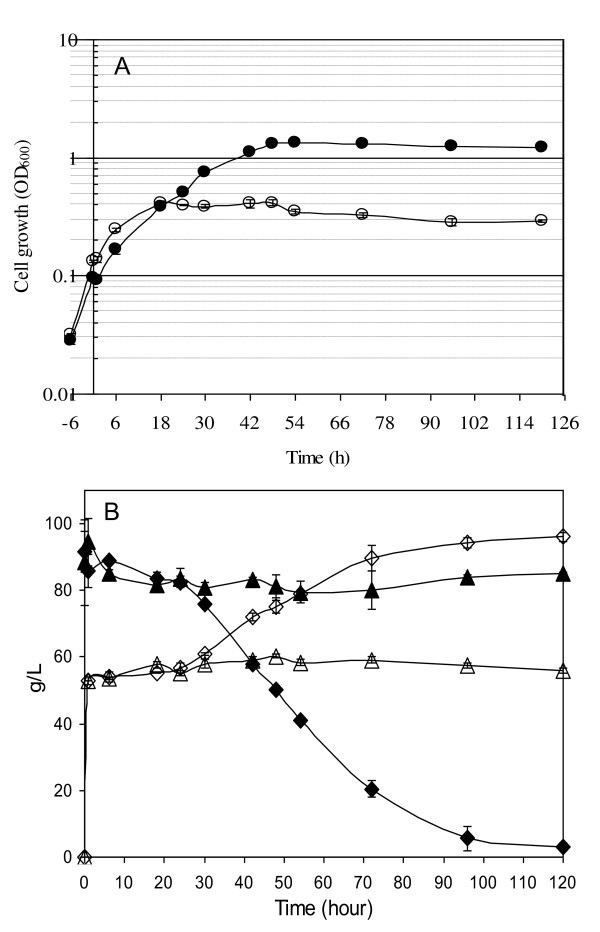
**Fermentation profiles under the ethanol stress**. Comparison of cell growth and ethanol conversion of *Saccharomyces cerevisiae *NRRL Y-50316 and NRRL Y-50049 over time in response to 8% (v/v) ethanol challenge on YM medium with 10% glucose. (A) Cell growth as measured by OD_600 _for Y 50316 (●) and Y-50049 (○). (B) Mean values of glucose consumption (♦) and ethanol concentration (◊) for Y-50316 versus glucose (▲) and ethanol (Δ) for Y-50049.

With the addition of ethanol at 8% (v/v) 6 h after inoculation, yeast growth of the two strains showed a similar OD reading briefly followed by an obvious separation after 18 h between the ethanol-tolerant strain Y-50316 and its parental strain Y-50049. Strain Y-50316 exhibited a continued growth through a log phase in 48 h to reach an OD_600 _reading of 1.3 when the ethanol concentration was 75.1 g/L (9.5%, v/v) (Figure 3A and 3B). On the other hand, Y-50049 ceased growth since 18 h and apparently went into cell lysis stages and never recovered. Consequently, no glucose consumption and ethanol conversion were observed for Y-50049 under the ethanol challenge (Figure 3B). In contrast, the ethanol-tolerant strain Y-50316 displayed an accelerated glucose consumption and ethanol conversion after 24 h (Figure 3B). At 120 h, glucose was almost exhausted and the total ethanol concentration reached 96 g/L. Production of glycerol and acetic acid under the conditions of this study was insignificant (data not shown).

### Master equation for qRT-PCR Assays

**Figure 4:**
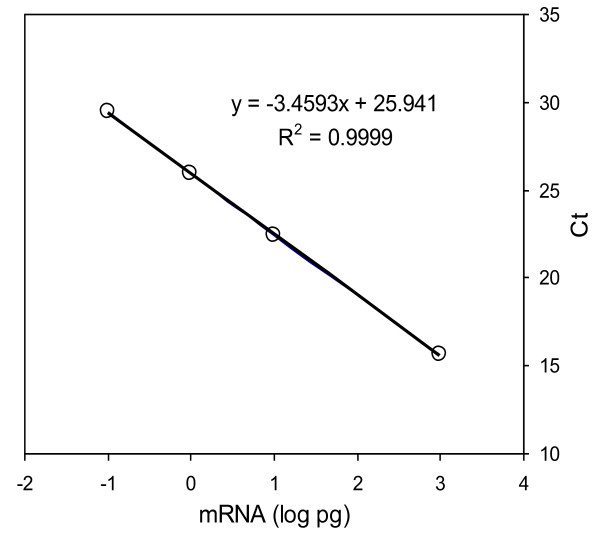
**Functional performance of universal RNA controls for real time qRT-PCR assays**. Robust calibration control genes of *MSG*, *CAB*, *RBS1*, and *ACTB *at 0.1, 1, 10, and 1,000 pg over 80 individual 96-well reaction plates for *Saccharomyces cerevisiae *NRRL Y-50316 and NRRL Y-50049 treated with 8% (v/v) ethanol demonstrated highly fitted linear relationship between the mRNA input (log pg) and PCR cycle numbers (Ct) by a master equation for assays on ABI 7500 real time PCR System. Standard deviation of the slope and the intercept of the master equation based on 80 individual standard curves under varied experimental conditions was 0.0458 and 0.0966, respectively.

Using *CAB *as a sole reference to set a manual threshold at 26 Ct for data acquisition (see methods) [40], raw data were normalized and analyzed for the entire PCR reactions applied in 80 individual 96-well plate runs. As anticipated, extremely high levels of consistent performance was obtained for the universal control genes as a calibration standard (Table 2). A standard curve was constructed for all individual plate reactions applying the universal control genes *MSG*, *CAB*, *RBS1*, and *ACTB *(Additional File 1). A highly fitted master equation was established (Figure 4) using the pooled data for all reference control reactions as follows:(1)

**Table 2:** Robust performance of standard control genes using *CAB *as sole reference to set a manual threshold at 26 Ct and a master equation derived from 80 replicated plate reactions on Applied Biosystems 7500 real time PCR System

Control gene	Reference Ct	Mean Ct	Stdev	Estimated mRNA (pg)	Input mRNA (pg)	Consistency (%)
*MSG*		29.429	0.077	0.098	0.1	98.1
*CAB*	26.0	25.965	0.037	0.984	1	98.4
*RBS1*		22.388	0.019	10.64	10	93.6
*ACTB*		15.604	0.019	973.25	1000	97.3

where X represents mRNA (log pg) and Y equals qRT-PCR cycle number (Ct) estimated for all reactions performed on an ABI 7500 real time PCR System. Average PCR amplification efficiency for the entire reaction set was 95% (data not shown) as measured by the slope of the standard curves [40,46].

### Enriched background of gene transcription abundance

**Figure 5:**
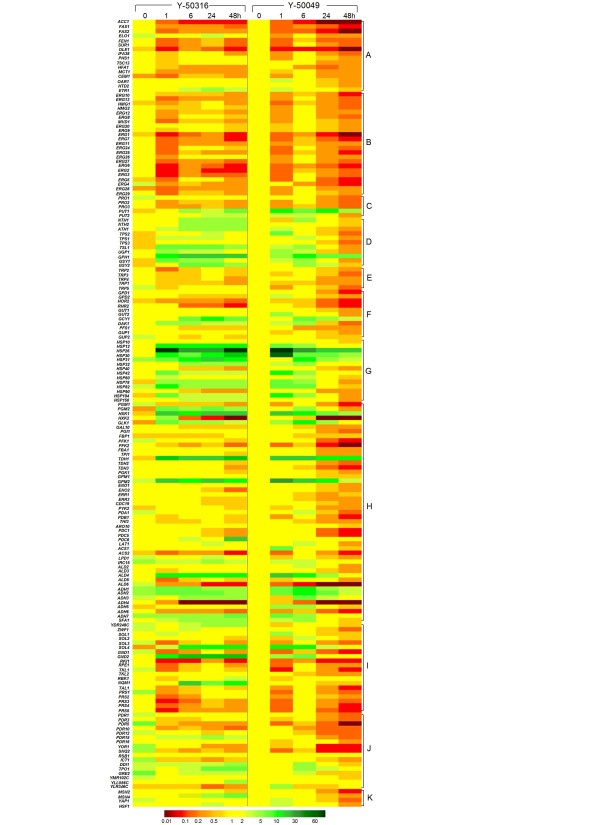
**Gene expression under the ethanol stress**. Comparison of mRNA expression of *Saccharomyces cerevisiae *NRRL Y-50316 and NRRL Y-50049 by fold changes from 0 h to 48 h after the ethanol challenge treatment. Corresponding genes were categorized by functions involved in fatty acid biosynthesis (A), ergosterol metabolism (B), proline metabolism (C), trehalose metabolism (D), tryptophan metabolism (E), glycerol metabolism (F), heat shock protein family (G), glycolysis (H), pentose phosphate pathway (I), pleiotropic drug resistance gene family (J) and related transcription factor genes (K). Expressions for each gene at each time point were presented in relative fold changes against that of Y-50049 at 0 h. Green indicates enhanced expression, red for repressed expression, and yellow for no significant changes. Scales of expressions were indicated by a an integrated color bar at the bottom.

For ethanol-tolerant strain Y-50316, initial mRNA abundance of many genes showed significant difference without ethanol challenges compared with its parental strain Y-50049 under the same growth conditions. At the designated 0 h, a time point the culture was incubated for 6 h before the ethanol addition, at least 35 genes were found having higher gene transcription abundance for the ethanol-tolerant yeast than its parental strain (Figure 5 and Table 3). In this group, 26 were first identified as ethanol tolerance related genes as follows: *ELO1, GUP2, HSP31, PGM1, PFK1, PDA1, LPD1, IRC15, ADH2, ADH3, ADH7, ZWF1, SOL3, GND1, PRS1, PDR1, PDR5, PDR12, YOR1, SNQ2, ICT1, DDI1, TPO1, GRE2, YDR248C*, and *YMR102C *(Table 3). Since the higher levels of transcripts were acquired through the tolerant adaptation procedures, these genes are considered as ethanol-tolerance related. They belong to groups of heat shock proteins, glycolysis, pentose phosphate pathway, fatty acid metabolism and the PDR gene family. The increased abundance for many genes was significantly higher in Y-50316 as compared with its parental strain Y-50049, especially for those in PDR gene family such as *PDR5, YOR1, SNQ2*, and* GRE2.*

**Table 3:** Functional categories and comparative expression fold changes of candidate and key genes for ethanol tolerance and ethanol fermentation for tolerant *Saccharomyces cerevisiae *NRRL Y-50316 and its parental strain Y-50049 over time under the ethanol challenge

Gene and Category	Function description	Y-50316					Y-50049					Msn4p/Msn2p	Yap1p	Hsf1p	Pdr1p/Pdr3p
		0 h	1 h	6 h	24 h	48 h	0 h	1 h	6 h	24 h	48 h				
Heat shock proteins															
*HSP12*	Plasma membrane localized heat shock protein	0.7	**5.2**	**7.8**	**6.7**	**5.6**	1.0	**4.3**	**2.1**	1.3	1.2	7	0	1	0
*HSP26*	Small heat shock protein with chaperone activity	0.9	**55.2**	**30.0**	**31.7**	**54.4**	1.0	**59.5**	**34.8**	**17.8**	**15.3**	4	0	7	0
*HSP30*	Hydrophobic plasma membrane localized heat shock protein	1.0	**7.6**	**3.3**	**7.1**	**23.9**	1.0	**48.8**	**4.6**	**3.2**	**3.0**	0	3	0	0
HSP31*	Member of the DJ-1/ThiJ/PfpI superfamily, chaperone and cysteine protease	**2.1**	**3.6**	**7.9**	**10.2**	**9.3**	1.0	1.3	**5.5**	**2.1**	**1.8**	1	2	4	0
***HSP32***	Possible chaperone and cysteine protease	0.8	1.0	**2.4**	**2.1**	**2.3**	1.0	**1.5**	**2.1**	1.4	1.0	4	0	6	0
*HSP42*	Small heat shock protein with chaperone activity	0.8	**3.8**	**1.5**	**1.6**	**1.6**	1.0	**6.9**	**2.8**	1.2	0.7	3	0	8	0
*HSP78*	Heat shock protein of ATP-dependent proteases, mitochondrial	0.6	**3.0**	**2.2**	**2.8**	**2.9**	1.0	**4.3**	**2.0**	0.9	0.3	3	1	8	0
HSP82*	Heat shock protein,Hsp90 chaperone required for pheromone signaling	**1.7**	**7.6**	**2.6**	**2.2**	**2.4**	1.0	**3.4**	**3.4**	1.3	0.6	2	1	4	0
*HSP104*	Heat shock protein	0.5	**3.7**	**1.6**	**1.7**	**1.9**	1.0	**8.8**	**2.6**	1.0	0.4	3	1	10	0
***HSP150***	O-mannosylated heat shock protein	1.4	1.0	**1.9**	**1.7**	**1.7**	1.0	1.0	1.0	0.7	0.4	2	1	0	0

Trehalose and glycogen metablism															
PGM1*	Phosphoglucomutase, minor isoform	**1.6**	0.6	0.6	0.6	0.4	1.0	0.4	0.7	0.3	0.2	3	0	2	0
*PGM2*	Phosphoglucomutase, major isoform	0.4	**3.6**	**2.6**	**3.8**	**2.3**	1.0	1.4	**2.4**	0.9	0.5	7	1	0	0
*UGP1*	UDP-glucose pyrophosphorylase	1.1	**2.4**	**1.5**	**1.9**	1.2	1.0	**2.6**	**1.5**	0.6	0.3	5	0	2	0
*GPH1*	Glycogen phosphorylase	1.0	**5.2**	**14.3**	**19.9**	**17.7**	1.0	**2.4**	**6.6**	**4.5**	**3.5**	3	1	0	0
*GSY1*	Glycogen synthase	0.6	**3.4**	**2.2**	**2.0**	1.0	1.0	**1.6**	**2.5**	1.1	0.5	2	0	0	0
***GSY2***	UDP-glucose--starch glucosyltransferase	0.6	1.2	**3.2**	**3.2**	**2.4**	1.0	1.4	**2.1**	**1.5**	0.6	2	1	4	0
*TSL1*	alpha-trehalose-phosphate synthase	0.6	**3.2**	**3.5**	**3.1**	**2.3**	1.0	**1.8**	**2.3**	1.1	0.4	7	0	7	0
*TPS1*	alpha-trehalose-phosphate synthase	0.6	**1.5**	**1.9**	**1.9**	1.1	1.0	1.3	**1.7**	0.7	0.4	6	2	2	0
*TPS3*	Regulatory subunit of trehalose-6-phosphate synthase/phosphatase complex	0.7	0.7	0.9	1.1	0.9	1.0	0.8	1.2	0.6	0.3	2	0	2	0
*ATH1*	Acid trehalase, vacuolar	1.1	**1.6**	**2.1**	**2.2**	**2.0**	1.0	**1.7**	1.2	0.6	0.4	1	1	4	0
*NTH1*	Neutral trehalase	0.9	1.3	**2.3**	**2.7**	**2.5**	1.0	0.6	**2.0**	1.2	0.5	3	0	2	0
*NTH2*	alpha-trehalase	1.0	1.4	**2.1**	**2.8**	**2.8**	1.0	0.9	1.4	0.9	0.5	1	1	2	0

Glycolysis															
*HXK1*	Hexokinase I	0.5	**16.8**	**6.9**	**13.1**	**15.8**	1.0	**14.1**	**8.1**	**3.8**	**2.2**	5	0	4	0
*GLK1*	Glucokinase	0.4	**4.0**	**2.7**	**2.4**	**1.8**	1.0	**2.5**	**6.3**	**2.3**	0.8	4	0	0	0
*PGI1*	Glycolytic enzyme phosphoglucose isomerase	1.4	0.8	0.8	0.8	0.8	1.0	0.8	1.0	0.5	0.3	0	0	2	0
PFK1*	Alpha subunit of heterooctameric phosphofructokinase involved in glycolysis	**1.6**	0.9	0.8	0.7	0.5	1.0	0.9	1.3	0.3	0.2	0	0	2	0
*FBA1*	Fructose 1,6-bisphosphate aldolase	1.2	1.0	0.8	0.9	0.7	1.0	0.9	1.0	0.4	0.3	0	1	1	0
*TDH1*	Glyceraldehyde-3-phosphate dehydrogenase 1	0.6	**25.8**	**16.4**	**17.8**	**20.2**	1.0	**11.4**	**17.3**	**9.8**	**5.9**	2	2	0	0
*TDH2*	Glyceraldehyde-3-phosphate dehydrogenase 2	1.3	1.3	1.0	0.7	0.5	1.0	1.1	1.1	0.4	0.2	0	0	0	0
*TDH3*	Glyceraldehyde-3-phosphate dehydrogenase 3	1.1	0.9	0.8	0.7	0.4	1.0	0.8	0.6	0.2	0.2	3	2	1	0
GPM2*	Homolog of Gpm1p phosphoglycerate mutase	**1.6**	**10.4**	**6.1**	**10.2**	**5.6**	1.0	**34.6**	**16.9**	**5.2**	**1.8**	1	3	4	0
*ERR1*	Enolase related protein	0.9	1.1	1.0	0.8	0.9	1.0	1.1	0.6	0.4	0.5	4	0	4	0
*PYK2*	Pyruvate kinase	0.7	0.9	0.9	0.9	0.5	1.0	0.5	1.1	0.5	0.3	1	1	0	0
IRC15*	Putative dihydrolipoamide dehydrogenases	**2.1**	**1.9**	**1.6**	**2.2**	**1.8**	1.0	**2.0**	**1.6**	1.2	0.8	2	1	2	0
LPD1*	Dihydrolipoamide dehydrogenase	**1.5**	0.7	1.0	**1.7**	1.3	1.0	0.7	1.2	0.6	0.4	2	3	0	2
PDA1*	E1 alpha subunit of the pyruvate dehydrogenase (PDH) complex	**1.9**	0.8	1.2	1.2	0.9	1.0	0.7	**1.7**	0.6	0.3	2	1	2	0
*ALD4*	Mitochondrial aldehyde dehydrogenase, utilizes NADP+ or NAD+ equally as coenzymes	0.9	**5.3**	**7.8**	**7.0**	**6.1**	1.0	**11.3**	**5.3**	**2.8**	1.4	3	3	0	0
ALD6*	Cytosolic aldehyde dehydrogenase	**1.9**	0.4	0.4	0.2	0.1	1.0	0.3	0.1	0.1	0.1	4	1	0	2
ADH1*	Alcohol dehydrogenase I	**2.9**	**4.2**	**4.0**	**2.9**	**2.0**	1.0	**4.3**	**5.8**	**2.5**	**1.8**	4	1	2	0
ADH2*	Alcohol dehydrogenase II	**2.9**	**4.4**	**4.8**	**3.9**	**2.4**	1.0	**4.8**	**7.1**	**3.4**	**1.9**	2	0	2	0
ADH3*	Alcohol dehydrogenase III	**2.0**	0.8	**2.5**	**2.6**	**2.3**	1.0	0.6	**4.0**	**1.7**	1.0	0	1	0	0
ADH7*	NADP(H)-dependent alcohol dehydrogenase	**2.9**	**2.6**	**2.3**	**2.4**	**3.2**	1.0	**3.9**	**2.9**	1.4	1.1	1	2	2	0
***SFA1***	Long-chain alcohol dehydrogenase	1.2	**1.7**	**2.0**	**2.4**	**2.3**	1.0	**1.9**	**2.3**	1.0	0.6	1	0	2	0

Pentose phosphate pathway															
ZWF1*	Glucose-6-phosphate dehydrogenase	**1.8**	1.2	**1.5**	1.3	0.9	1.0	0.8	1.2	0.7	0.3	5	1	0	0
YDR248C*	Sequence similarity to bacterial and human gluconokinase	**1.7**	0.7	**1.5**	**3.0**	**2.4**	1.0	0.7	1.4	0.7	0.5	3	1	0	0
SOL3*	Possible 6-phosphogluconolactonase	**1.8**	0.3	0.6	1.3	0.4	1.0	0.4	0.9	0.4	0.3	1	3	0	0
***SOL4***	putative 6-phosphogluconolactonase	0.3	**1.8**	**8.2**	**9.9**	**7.5**	1.0	**6.7**	**7.0**	**1.5**	1.1	1	0	6	0
GND1*	6-phosphogluconate dehydrogenase	**1.8**	0.3	0.3	0.9	0.5	1.0	0.3	0.6	0.3	0.1	1	0	0	0
***GND2***	6-phosphogluconate dehydrogenase	0.9	**8.6**	**23.1**	**26.2**	**23.0**	1.0	**2.1**	**4.0**	1.2	1.0	3	1	7	0
***NQM1***	Transaldolase of unknown function	1.1	0.8	**10.2**	**3.4**	**6.1**	1.0	1.2	1.1	0.6	0.6	3	1	2	0
TKL1*	Transketolase 1	**1.6**	0.2	0.6	1.0	0.6	1.0	0.2	0.8	0.3	0.1	1	1	2	0
*TKL2*	Transketolase 2	0.9	0.8	1.3	0.7	1.1	1.0	1.0	0.5	0.5	0.5	2	2	1	0
PRS1*	5-phospho-ribosyl-1(alpha)-pyrophosphate synthetase	**2.2**	0.3	0.5	1.0	0.9	1.0	0.3	1.1	0.4	0.3	0	2	6	0

PDR family															
PDR1*	zinc finger transcription factor for pleiotropic drug response	**1.7**	0.9	1.0	0.9	1.0	1.0	0.7	1.0	0.4	0.3	0	1	0	0
PDR5*	Plasma membrane ATP-binding cassette (ABC) transporter	**4.4**	0.5	0.4	0.3	0.4	1.0	0.2	0.6	0.3	0.1	1	2	6	8
PDR12*	Plasma membrane ATP-binding cassette (ABC) transporter	**1.5**	1.3	0.7	0.7	0.9	1.0	1.0	0.6	0.3	0.2	0	1	2	0
***PDR15***	ATP binding cassette (ABC) transporter of the plasma membrane	1.3	**1.7**	**1.5**	**2.3**	**1.7**	1.0	1.0	0.9	0.4	0.3	5	0	0	3
YOR1*	ATP binding cassette (ABC) transporter of the plasma membrane	**2.2**	0.8	0.8	0.5	0.4	1.0	0.6	0.9	0.1	0.1	2	1	0	2
SNQ2*	ATP binding cassette (ABC) transporter of the plasma membrane	**2.3**	0.6	0.4	0.7	0.5	1.0	0.3	0.5	0.2	0.1	1	2	0	7
ICT1*	Lysophosphatidic acid acyltransferase	**2.0**	0.6	0.6	0.4	0.6	1.0	1.0	1.2	0.7	0.4	1	0	2	2
DDI1*	DNA damage-inducible v-SNARE binding protein	**1.7**	**1.7**	**2.0**	**1.7**	**2.4**	1.0	1.1	**2.0**	1.0	0.6	1	1	0	0
TPO1*	Vacuolar polyamine-H+ antiporter	**1.7**	1.0	**2.0**	**3.1**	**3.5**	1.0	1.4	**2.6**	**1.9**	1.0	2	3	0	2
GRE2*	Methylglyoxal reductase (NADPH-dependent)	**4.1**	1.4	**1.5**	**1.6**	**1.8**	1.0	1.3	**1.5**	**0.6**	0.5	0	1	2	2
YMR102C*	Protein of unknown function	**1.6**	1.2	1.1	1.2	1.0	1.0	1.2	0.9	0.7	0.6	1	0	0	3

Fatty acid metabolism															
***ETR1***	Mitochondrial respiratory function protein	0.9	1.0	**1.5**	**2.1**	**1.7**	1.0	**1.6**	1.3	0.7	0.5	2	2	2	0
ELO1*	Elongase I, Fatty acid elongation protein	**1.6**	0.8	1.3	**1.8**	1.0	1.0	0.5	0.7	0.4	0.3	0	1	2	0
*HTD2*	Mitochondrial 3-hydroxyacyl-thioester dehydratase involved in fatty acid biosynthesis	1.1	0.9	1.1	1.1	1.0	1.0	0.7	1.1	0.5	0.5	0	0	0	0

Egosterol biosynthesis															
ERG4*	C-24(28) sterol reductase	**1.5**	0.5	0.6	0.5	0.3	1.0	0.7	0.4	0.2	0.2	0	0	2	2
*ERG20*	Farnesyl-pyrophosphate synthetase	0.9	0.7	0.9	0.9	0.6	1.0	0.6	1.3	0.6	0.4	1	1	0	0
*ERG26*	C-3 sterol dehydrogenase	1.0	0.4	0.9	0.8	0.8	1.0	0.4	0.8	0.5	0.4	0	1	5	0

Proline metabolism															
*PUT1*	Proline oxidase	0.6	0.8	**2.7**	**1.8**	**4.9**	1.0	**5.1**	**3.8**	**6.0**	**2.6**	0	0	0	0
PRO1*	Gamma-glutamyl kinase, catalyzes the first step in proline biosynthesis	**1.6**	1.0	0.7	0.9	0.7	1.0	0.7	1.0	0.5	0.3	0	0	2	0

Tryptophan biosynthesis															
TRP5*	Tryptophan synthase	**1.5**	0.5	1.0	1.4	0.7	1.0	0.4	1.3	0.5	0.2	4	2	0	0

Glycerol metabolism															
*DAK1*	Dihydroxyacetone kinase	1.2	**2.2**	**2.0**	**1.9**	**1.8**	1.0	**1.6**	**2.0**	0.7	0.3	0	0	0	0
***GCY1***	Putative NADP(+) coupled glycerol dehydrogenase	1.1	0.9	**4.3**	**5.4**	**4.8**	1.0	1.1	**4.1**	**2.2**	**1.7**	1	1	2	0
*GPD1*	NAD-dependent glycerol-3-phosphate dehydrogenase	1.3	0.8	1.0	1.1	0.5	1.0	1.4	1.0	0.3	0.2	4	1	0	0
*GUP1*	Multimembrane-spanning protein essential for proton symport of glycerol	1.2	1.0	0.9	1.2	0.8	1.0	0.6	1.0	0.5	0.3	0	0	0	0
GUP2*	Putative glycerol transporter involved in active glycerol uptake	**1.8**	0.8	0.6	1.0	0.6	1.0	0.7	1.0	0.6	0.5	1	0	0	0

Transcription factors															
*MSN2*	Transcriptional activator related to Msn4p	1.0	0.8	0.7	0.8	0.5	1.0	1.2	0.7	0.4	0.2	1	0	2	0
*MSN4*	Transcriptional activator related to Msn2p	1.0	0.8	1.3	**2.5**	**3.2**	1.0	1.0	0.7	0.5	0.4	4	0	2	0
YAP1*	Transcriptional activator involved in oxidative stress response	**1.5**	0.9	0.8	1.0	0.7	1.0	**1.7**	1.0	0.5	0.3	1	2	2	0
*HSF1*	Heat shock transcription factor	1.4	1.3	1.2	**1.5**	1.3	1.0	**1.6**	1.1	0.7	0.4	1	3	2	0

* Genes showing significantly enriched transcription abundance in Y-50316 prior to ethanol challenge (p < 0.01).

Genes in bold indicate new reports by this study and the expression fold changes in bold indicate an increase of greater than 1.5-fold (p < 0.01) compared with a wild type control.

Numbers of protein binding motifs related to transcription factors Msn4p/Msn2p, Yap1p, Hsf1p and Pdr1p/Pdr3p for each gene were marked under each transcription factor

### Transcription dynamics of heat shock protein genes

**Figure 6:**
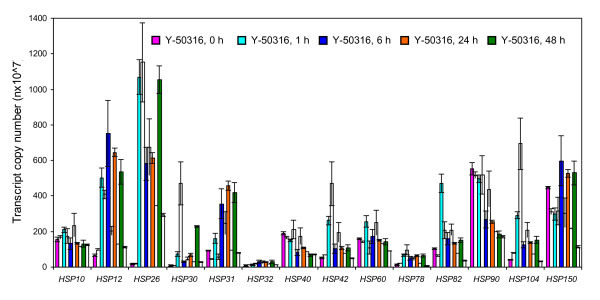
**Quantitative expression of heat shock protein genes**. Comparisons of transcription expressions in gene copy numbers (nX10^7^) for heat shock protein genes between ethanol-tolerant strain *Saccharomyces cerevisiae *NRRL Y-50316 and its parental strain NRRL Y-50049 under the ethanol challenge over time. Mean values are presented with error bars of standard deviations. Values at different time points are presented by a specific colored bar as shown in legends for the tolerant Y-50316 and an immediately adjacent open bar on its right for its parental strain Y-50049 of the same time point.

All 14 examined heat shock protein genes demonstrated normal or enhanced expressions at the earlier stage, such as at 1 or 6 h after ethanol challenge for both strains (Figure 5 and 6). However, most heat shock protein genes in Y-50049 were repressed at 24 and 48 h and only three genes, *HSP26, HSP30 *and *HSP31*, remained induced for the parental strain Y-50049. But the expression abundance of these genes was significantly less than that of the ethanol-tolerant strain Y-50316 (Table 3). Y-50316, on the other hand, had 10 genes, *HSP12*, *HSP26*, *HSP30*, *HSP31*, *HSP32, HSP42, HSP78*, *HSP82*, *HSP104*, and *HSP150 *showing significantly induced expressions from 24 to 48 h. Among these, *HSP26 *displayed the highest expression levels at all time points. Except for *HSP40 *and *HSP90*, all other heat shock protein genes of Y-50316 had distinct increased expression dynamics over time compared with its parental strain Y-50049 (Additional File 2). For example, *HSP31 *and *HSP82 *in Y-50316 were highly expressed at each time point. These heat shock proteins were found to be involved in cellular structure-function relationships at multiple locations including nucleus, mitochondrion, cytoplasm, cytoskeleton, membrane, and cell wall (Additional File 3).

### Adaptive expressions of trehalose and glucose metabolism genes

Although the initial transcription abundance was low, all examined trehalose and glycogen metabolism genes responded positively to the ethanol challenge over time. Transcription levels of the 11 trehalose metabolism genes in Y-50316 were consistently enhanced from 1 to 48 h, especially for *NTH1*, *NTH2*, *ATH1, TSL1, TPS1, GPH1*, and* GSY2 *(Figure 5, Table 3 and Additional File 2). *GPH1*, a gene involved in glycogen catabolism had almost 20-fold increased transcription abundance, the highest level in this group at 24 h for the tolerant Y-50316. Its expression levels were significantly greater at every time point compared with those of the parental strain (Table 3). *GSY2 *encoding for UDP-glucose-starch glucosyltransferase, another highly induced expressed gene in Y-50316, was identified as a new candidate gene for ethanol tolerance. For the parental strain Y-50049, most genes in this group had similar induced response at 1 and 6 h after the ethanol challenge. However, except for *GPH1*, all other 10 genes were reversed as repressed after 6 h.

Transcription dynamic response was more complex for genes involving in glycolysis and pentose phosphate pathways. Many genes in this group demonstrated persistent high abundant expressions from 1 to 48 h after the ethanol challenge such as *PGM2, HXK1, GLK1, TDH1, GPM2, IRC15, ALD4, ADH1, ADH2, ADH3, ADH7, SFA1, SOL4, GND2, NQM1*, and *YDR248C *(Figure 5 and Table 3). Especially for *GND2, TDH1 *and *NQM1*, their expression levels were constantly higher at all time points. The expression patterns of most genes in this group in Y-50316 were distinct from that of its parental strain Y-50049, particularly after 6 h when many genes of the latter were significantly repressed. In addition to genes with enriched transcriptional abundance, at least another seven previously unreported genes in this group were identified as new candidate genes for ethanol-tolerance and ethanol production under the stress including *ADH7, SFA1*, *GND2, NQM1*, *SOL4, IRC15*, and *YDR248C *(Table 3).

**Figure 7:**
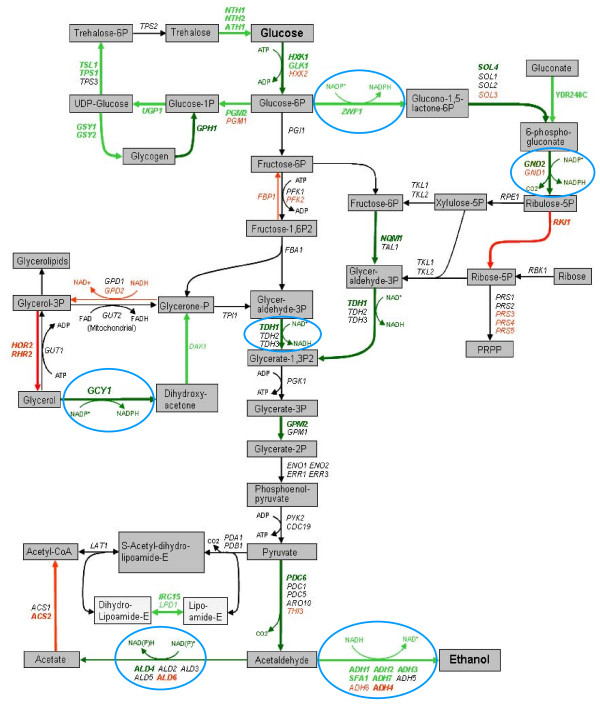
**Glucose metabolic pathway response**. Illustrative pathways of ethanol- and inhibitor-tolerant mutant *Saccharomyces cerevisiae *NRRL Y-50316 involved in trehalose-glycolysis-pentose phosphate pathway in response to ethanol challenges inferred by dynamic quantitative mRNA expression analysis and metabolic profiling analysis compared with its parental strain NRRL Y-50049. Dark green arrowed lines and letters indicate high levels (5.1-60 fold increase for at least one critical time point) of mRNA expression and enhanced pathways, green for significant levels (1.5-5 fold increase for at least one critical time point) of enhanced transcription and pathways; black indicates normal or nearly normal levels of transcription and pathway events, red for repressed expression, reactions, or pathways. Bold lines and letters indicate the levels of expression and pathways are statistically significant at* P *< 0.05. Reactions involved in NAD(P)H regeneration steps are circled in blue.

Many important genes in this group displayed a normal or non induced expressions under the ethanol challenge for the tolerant Y-50316 such as *PGI1, PFK1, FBA1, TDH2, TDH3, TPI1, PGK1, GPM1, ENO1, EBO2, ERR1, ERR3, PYK2, CDC19, PDC1, PDC5, ARO10, THI3, ALD2, ALD3, ADH5, PDA1, PDB1, ACS1, SOL1, SOL2, TKL1*, and *TKL2 *(Figure 7, Table 3 and Additional File 2). In contrast, for the parental Y-50049, most of these genes were repressed at the lower levels especially after 6 h (Figure 5). The transcript of *ZWF1 *in Y-50316 was not only enriched initially, but constantly displayed greater levels of expression at every time point compared with its parental Y-50049 (Table 3). Some enhanced genes in the tolerant Y-50316 are involved in multiple functions of carbohydrate metabolism and mitochondrion functions such as *HXK1, GLK1, GND2, TDH1, SOL4, GPM2, ADH1*, and *ALD4 *(Additional File 3).

### Enhanced expressions of PDR gene family

Seventeen genes in this group were selected based on our preliminary tests of yeast stress tolerance. Among which, 13 genes were identified as candidate genes closely related to ethanol tolerance by enriched background of transcription abundance, increased, normal or recoverable expressions under ethanol challenge as demonstrated by the tolerant Y-50316 (Table 3 and Additional File 2). *PDR15*, *DDI1, TPO1*, and* GRE2 *maintained noticeable higher levels of expressions at all time points in addition to their enriched mRNA abundance at 0 h for Y-50316. Other genes in this group such as *PDR1, PDR16*, *YMR102C*, *PDR3, PDR5, PDR12, PDR16, YOR1*, and *SNQ2 *for Y-50316 were expressed at normal levels or recoverable at later stages. On the other hand, these genes in Y-50049 were repressed.

### Comparative expressions of transcription factor genes

**Figure 8:**
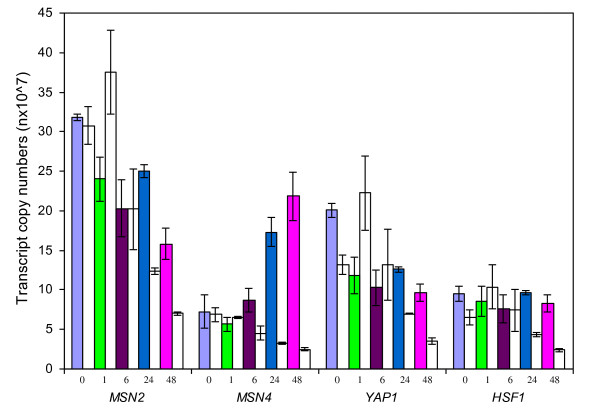
**Expression response of transcription factor genes**. Comparisons of transcription expressions in gene copy numbers (nX10^7^) for transcription factor genes between ethanol-tolerant *Saccharomyces cerevisiae *NRRL Y-50316 and its parental strain NRRL Y-50049 under the ethanol challenge over time. Mean values are presented with error bars of standard deviations. Values at different time points are presented by a specific colored bar as shown in legends for the tolerant Y-50316 and an immediately adjacent open bar on its right for the parental strain Y-50049 at the same time point.

In addition to the *PDR1 *and *PDR3 *expressions representing Pdr1p and Pdr3p described above, four other genes encoding transcription factors Msn4p, Msn2p, Yap1p and Hsf1p showed distinct expression patterns over time between the two strains. Expression levels of these four genes in Y-50049 were constantly reduced with the time exposed to ethanol (Figure 8). For the tolerant Y-50316, *MSN2, YAP1 *and *HSF1 *represented a similar type of expressions that was moderately repressed at 1 and 6 h after exposure to ethanol (Figure 8). At 24 h, their expression levels were remarkably increased and significantly greater in Y-50316 than those in Y-50049. At 48, although significantly higher than the parental strain, transcription levels of these three genes in Y-50316 decreased. *MSN4*, on the other hand, displayed a unique type of continued increase of up-regulated expressions from 1 to 48 h. At the critical time point of 6 h, unlike the other three repressed genes, *MSN4 *expression in Y-50316 was consistently increased from the previous time point, significantly higher than the parental control (Figure 8 and Table 3). This consistent increase of transcription abundance was distinct and observed at 48 h again for *MSN4 *in Y-50316.

### Transcriptional regulation under ethanol stress

**Figure 9:**
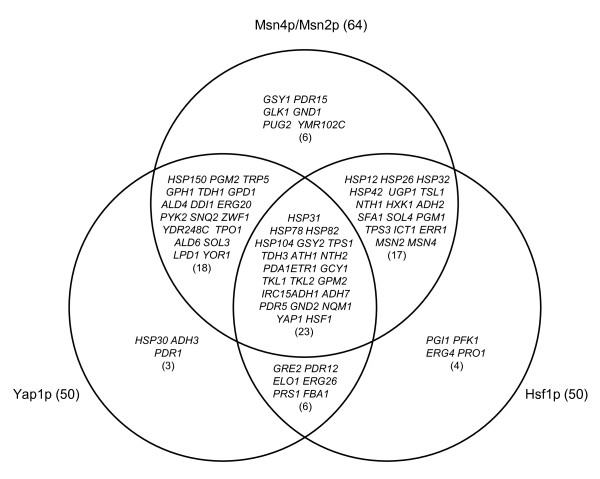
**Shared protein binding motifs of candidate genes**. A Venn diagram showing shared common protein binding motifs of transcription factors Msn4p/Msn2p, Hsf1p, and Yap1p in their promoter regions for 82 candidate and key genes for ethanol tolerance and subsequent ethanol fermentation under ethanol stress in yeast.

Most members of PDR gene family were found to have protein binding motifs of transcription factor Pdr1p/Pdr3p in their promoter regions (Table 3). Significantly up-regulated *PDR15, TPO1, GRE2 *and *YMR102C *had at least two binding motifs. Several genes in other functional categories also shared the Pdr1p/Pdr3p binding site. The number of protein binding motifs of transcription factors Msn4p/Msn2p, Yap1p and Hsf1p for the ethanol tolerance candidate genes was remarkably large. Among 82 candidate genes of ethanol tolerance identified in this study, 77 genes were found to have a protein binding motif of Msn4p/Msn2p, Yap1p or Hsf1p; and 23 genes shared the common binding sequence for all of the three transcription factors (Figure 9 and Table 3). The four newly identified ethanol-tolerant candidate genes *HSP31, HSP32*, *HSP150 *and *GND2 *by this study were found to share the same transcription factor Msn4p/Msn2p. *GND2, HSP31 *and *HSP32 *also appeared co-regulated by Hsf1p, and *GND2, HSP31 *and *HSP150*, by Yap1p.

### Expression responses of other genes

Expression levels of gene transcripts involved in fatty acid metabolism were generally low and repressed for both strains in response to the ethanol challenge except for *ELO1, ETR1*, *PHS1, TSC13, OAR1*, and* HTD2 *in Y-50316 having induced or recoverable expressions (Figure 5 and Table 3). Similarly, most genes in ergosterol metabolism group were repressed but *ERG20, ERG24 *and *ERG26 *in tolerant Y-50316 appeared to have normal or recoverable transcription expression potential over time (Figure 5 and Table 3). While all five tryptophan biosynthesis genes in parental Y-50049 were repressed over time, *TRP5 *in the tolerant Y-50316 was able to withhold the ethanol challenge (Table 3). Other four genes were mostly less repressed in Y-50316 than in Y-50049 (Additional File 2). Among five proline biosynthesis genes, *PUT1 *was induced for both strains. Expression patterns of most glycerol metabolism genes under ethanol challenge were similar for both strains with a few exceptions of Y-50316 genes including *DAK1*, *GCY1*, *GPD1, GUP2*, and *GUP1*.

## Discussion

Applying a newly developed qRT-PCR array assays to unify gene expression data analysis, we demonstrated transcription expression dynamics for ethanol-tolerant mutant Y-50316 in response to ethanol challenge compared with its parental strain Y-50049 of *S. cerevisiae*. As opposed to a single "snapshot" observations, we used a more informative time-course design investigating selected gene expression response from initial (0 h), early growth (1 and 6 h), exponential/log phase (24 h), and entering stationary phase (48 h) relative to the cell growth stage under the ethanol challenge. The dynamics of gene expression over time closely correlated with metabolic profiles and cell growth phenotypes between the two strains. This allowed identification of at least 82 candidate and key genes for ethanol tolerance and subsequent ethanol fermentation under the ethanol stress. Among which, 36 genes were the first report by the present study. Our results also suggest a potential key regulatory role of Msn4p for ethanol-tolerance among other transcription factor and regulatory elements.

The newly developed data acquisition and analysis standard for qRT-PCR array assays using the robust mRNA as the PCR Ct reference provided reliable means to safeguard data fidelity and allowed unification of gene expression data for comparable analysis. Housekeeping genes are commonly used as quality controls for qRT-PCR but vary under different experimental conditions [42,47]. Among numerous systems developed [41-45], the universal RNA controls have been shown another successful applications under ethanol stress conditions in this study. An extended adaptation and applications of such methods for consistent quantitative gene expression analyses are expected in the future.

Genes associated with ethanol stress were mostly reported based on snapshots of gene expression response in yeast [11-13,15]. In this study, we investigated a time-course study comparing cell growth, viability, glucose-to-ethanol conversion, and gene expression dynamics for two closely related strains. This allowed assessment of phenotype associations and identification of legitimate candidate genes for ethanol tolerance. As demonstrated by this study, the parental strain showed briefly induced expression of numerous genes before becoming repressed and unable to establish a viable culture under the ethanol challenge. Uncovered by the expression dynamics of the tolerant strain, we are able to distinguish ethanol-tolerance candidate genes and tolerance-response from the transient stress-response in yeast. For example, unlike many heat shock protein genes in parental strain becoming repressed after 6 h, these genes in the tolerant Y-50316 showed continued inductions through 48 h. This indicated that the continued expression of those heat shock protein genes after 6 h is critical for the ethanol tolerance in yeast.

Heat shock proteins, mainly act as chaperones, insuring properly folding or refolding of nascent or denatured proteins and enzymes to maintain functional conformation [48-50]. For example, Hsp12p, Hsp26p, Hsp42p, Hsp78p, and Hsp82p were reported to prevent proteins from aggregating, and Hsp104p was able to disassemble protein aggregates that have accumulated in response to stress [51]. *HSP82*, a highly up-regulated gene in response to ethanol for the ethanol tolerant Y-50316 observed in our study, was reported to activate many key cellular regulatory and signaling proteins, such as transcription factors and regulatory kinases [49,50,52,53]. The lack of continued function of these genes and interactions with other relevant gene expression in Y-50049 led to no further metabolic functions. Recent proteomic studies suggested that mRNA is selectively processed and translated in stationary phase [16,54]. Our results of enhanced expressions of most heat shock protein genes at a relatively late stage such as 24 and 48 h, for the tolerant Y-50316 are supportive to this hypothesis.

In this study, we found three previously unreported heat shock protein genes, *HSP31, HSP32 *and *HSP150*, were highly enhanced in the tolerant Y-50316 and identified as candidate genes for the ethanol tolerance. Hsp31p and Hsp32p, functioning as a chaperone and cysteine protease, are involved in protein binding, peptidase and hydrolase activities. Significantly enhanced gene expressions of *HSP31 *and *HSP32 *in Y-50316 observed in this study suggests the potential involvement of Hsp31p and Hsp32p as chaperones against ethanol stress. In addition, *HSP31 *and *HSP32 *were found to have functions in cell component and biological process categories. Hsp150p is a protein involved in cell wall and structural molecule activity. Higher levels of transcription and continued expressions of *HSP150 *indicated its potential protective functions compared with its parental strain under the ethanol challenge. Many heat shock protein genes induced by ethanol stress are present in cytoplasm as well as in nucleus and mitochondrion [55]. Because up-regulated heat shock protein genes influence cell functions at multiple locations, this facilitates the functions of transcription factors in nucleus, improving ATP energy generation in metabolic processes, maintaining enzyme functions involving biosynthesis, catabolism, and ethanol production in cytoplasm.

The induced gene expressions related to trehalose and glycogen metabolism are expected to facilitate a stable intracellular environment under ethanol stress condition for survival and accelerated glucose metabolism. We found *GSY2*, a gene involved in glycogen biosynthesis and degradation was up-regulated over time as a new record. Since glycogen metabolism is very close to trehalose pathway, the two pathways likely affect each other. Storage carbohydrates such as trehalose are compatible solutes that can prevent cell dehydration and influx of excess salts into cells. Trehalose accumulation was observed under ethanol stress condition to reduce membrane permeability and proper folding of proteins [17,24,56]. Our findings of up-regulated *TPS1*, *TSL1*, *PGM2*, and *UGP1 *in this group were consistent with previously observed. Genes involved in trehalose degradation including *NTH1*, *NTH2*, and *ATH1 *were also induced by ethanol. These observations also agreed with previously reported [11,12,17,29]. Enhanced expression of trehalose degrading genes appeared to be necessary in order to balance trehalose concentration and energy required for cell functions [11,57].

As demonstrated in this study, rapid cell growth and highly integrated expression of genes involved in trehalose biosynthesis, glycolysis and pentose phosphate pathway were closely correlated for the ethanol-tolerant strain Y-50316. Continued enhanced expressions of many genes associated in these groups apparently contributed active energy metabolism (Figure 7). In addition, numerous genes able to maintain normal expressions in Y-50316 appeared to be important keeping gene interactive networks. These genes are necessary for the tolerant yeast to carry out the active metabolisms and complete the ethanol fermentation (Figure 7) while most of these genes were repressed for the parental strain Y-50049. The ethanol-tolerant Y-50316 was co-selected for inhibitor-tolerance derived from its parental Y-50049. Under the ethanol challenge, the ethanol-tolerant Y-50316 displayed tolerant gene expression dynamics leading to similar route of pathway activities especially in every cofactor regeneration step. Cofactor NADPH plays an important role in biosynthesis of amino acids, lipids, and nucleotides [58,59]. Under the ethanol stress condition described in this study, the glucose metabolic pathways also appeared having a well-maintained cofactor redox balance (Figure 7) as exampled for *GND2 *and *ZWF1 *in oxidative phase of pentose phosphate pathway, *ALD4 *in acetic acid production, and *GCY1 *in glycerol metabolism. Enhanced expression of *ZWF1*, *SOL4*, and *YDR248C *potentially provide sufficient substrate for a smooth pentose phosphate pathway flow. Therefore, sufficient NADPH supply likely contributes ethanol tolerance indirectly through efficient biosynthesis of amino acids, lipids, and nucleotides for cell growth and function. Similarly, *TDH1 *involved in NADH regeneration step was highly induced. The enhanced expressions of alcohol dehydrogenase genes *ADH1*, *ADH2*, *ADH3*, *ADH7*, and *SFA1*, together with other normally expressed genes in the intermediate steps of glycolysis, are critical to complete the fermentation.

For the above mentioned reasons, we consider tryptophan and proline synthesis genes *TRP5, PRO1*, and *PUT1 *as ethanol tolerance candidate genes. Our results support the involvement of these genes in ethanol-tolerance as suggested by previous studies [13,25,28]. Several genes involving in fatty acid metabolism were repressed except for *ETR1, ELO1 *and* HTD2 *having induced and normal expressions for the tolerant Y-50316. Ergosterol is another major component of cellular membranes that associated with maintenance of plasma membrane fluidity affecting ethanol tolerance [14,28]. Similarly with previous reported [11,12], most genes involved in ergosterol biosynthesis were repressed for both strains in this study. It is possible that the regulatory functions of the biosynthesis may not be significantly affected at transcriptional levels under the conditions of this study.

The PDR gene group is a new set of genes examined for ethanol tolerance in this study. Many PDR genes function as transporters of ATP-binding cassette proteins and are encoded for plasma membrane proteins that mediate membrane translocation of ions and a wide range of substrates. It impacts lipid and cell wall compositions and major facilitator superfamily proteins for cell detoxifications [60]. We previously found that PDR genes and regulatory elements played significant roles for tolerance and *in situ *detoxification of lignocellulose-derived inhibitors [61]. Since plasma membrane and cell walls are major targets of ethanol damages, we anticipated the involvement of these genes for reconditioning and remodeling membrane and cell walls in response to ethanol challenges. The significantly enriched background of transcriptional abundance and continuously increased expressions of several genes in this group for the ethanol tolerant yeast observed in this study support our hypothesis (Table 3).

The expressions of PDR genes are mainly controlled by transcription factor Pdr1p and Pdr3p [62]. As demonstrated in our study, many genes share the common transcription protein binding motif of Pdr1p/Pdr3p. Expressions of *PDR1 *in the tolerant Y-50316 was not significantly induced but constantly expressed at all time points compared with the parental strain. It needs to be pointed out that unless it is repressed, *PDR1 *does not have to be greatly induced to allow potential Pdr1p functions as a regulator [32,60]. We consider the ability of its expression under the stress is a tolerance response and suggest Pdr1p as a potential regulator involving the ethanol tolerance of Y-50316. As discussed above, genes able to express or recover to express normally under the stress are important to maintain gene interactions and cell functions. On the other hand, transcription factor genes *MSN4, MSN2, YAP1 *and* HSF1 *of the tolerant strains were highly abundance under the ethanol stress. Since many ethanol tolerance candidate genes sharing protein binding motifs of Msn4p/Msn2p, Yap1p and Hsf1p, these transcription factors are likely a core set of regulators for interactive expressions of ethanol tolerance. An *HSF1*-deletion mutant showed repressed expressions for its target genes usually induced by ethanol [63]. It has been demonstrated that Msn2p and Msn4p induces gene expression via a stress response element and triggers transcriptional response of the downstream genes [64,65]. Condition-specific roles in gene expression regulation by these transcription factors were also suggested [66]. Msn2p has been confirmed for its positive regulatory function of *HSP12 *and most heat shock protein genes for increased ethanol tolerance [67-69]. A double gene deletion *msn2msn4*-mutant showed hypersensitivity to environmental stress including higher ethanol concentrations [70]. We demonstrated that the increased expressions patterns of *MSN4 *overtime were distinct from other transcription factor genes. Our results suggest a potential key role of Msn4p in the dynamic response to the ethanol tolerance. However, limited information is available for Msn4p and further studies on its regulatory roles for tolerance are needed.

## Conclusion

The qRT-PCR array assay equipped with the robust mRNA reference and the master equation is an efficient means for quantitative gene expression analysis which unifies a large amount of expression data generated under different experimental conditions. The comparative characterizations of adaptive transcription dynamics for the two closely related strains are more informative and provide insight into dissection of mechanisms of ethanol tolerance. Analysis of the expression dynamics and association of other phenotypes allowed identification of candidate and key genes for the ethanol-tolerance and ethanol production under the stress. Enriched background of mRNA abundance of many genes appeared to be inheritable for the ethanol-tolerant yeast. Most ethanol-tolerance candidate genes were found sharing protein binding motifs of transcription factors Msn4p/Msn2p, Yap1p, Hsf1p and Pdr1p. The unique expression pattern of *MSN4 *in the ethanol-tolerant Y-50316 suggested a potential key regulatory role of Msn4p during the adaptive expression in yeast. Unlike repressed in the parental strain, genes able to maintain normal expressions under the ethanol-stress were necessary for the tolerant Y-50316 to function. Ethanol-tolerance candidate genes identified in this study are primarily associated with functional categories of cytoplasm, membrane, cell wall, response to stress, transportot, protein folding, oxidoreductase activity, protein binding and unknowns classified by gene ontology (GO). However, multiple functions and functions at multiple loci of many candidate genes are common. Ethanol induced genes are involved in at least 79 GO categories and every gene was found to have more than one function [55]. It's the time to revisit the traditional "one gene-one function" concept when evaluating gene regulatory networks. The complicated gene interactions cannot be overlooked in dissection of mechanisms of ethanol-tolerance in yeast.

## Methods

### Yeast strains, medium, and culture conditions

Ethanol-tolerant yeast *S. cerevisiae *NRRL Y-50316 and its inhibitor-tolerant parental strain NRRL Y-50049 (Agricultural Research Service Culture Collection, Peoria, IL, USA) were used in this study. Cultures were maintained and grown on a YM medium (3 g yeast extract, 3 g malt extract, and 5 g peptone, in 1 L distilled water) supplemented with 2 or 10% (w/v) glucose. Cultures were incubated on 300 ml medium in a fleaker system with agitation at 30°C as previously described [33]. A solid YM plate containing 2% agar was used to examine cell growth and viability. All experiments were carried out with two replications.

### Yeast adaptation and mutation selection

Adaptation procedures were developed based on procedures by Wei et al. [36] and Dinh et al. [27] with modifications. Briefly, inhibitor-tolerant strain NRRL Y-50049 was cultured on a YM with 10% glucose containing ethanol in designated concentrations. Cultures were treated with a quick freeze at -80°C at the mid-log phase and thawed at 30°C in a water-bath. The treatment procedures were repeated. Incubations were continued at 30°C until a stationary phase was reached. Surviving cultures were sequentially transferred to fresh medium containing higher ethanol concentrations. These procedures were repetitively carried out until a target tolerance level reached. Tolerant mutants were selected from at least 40 complete cycles using a medium containing no less than 8% ethanol. Culture characteristics were confirmed by cell morphology, growth rate, metabolic profiling, and sequence verification of its identity using nuclear large subunit ribosomal RNA gene [71].

### Assays for tolerance and viability

Cells were grown at 30°C and 250 rpm into the late exponential growth phase at OD_600 _reading of 1.0 when cultures contained approximately 1×10^7 ^cells/ml. An assay using serial dilutions of the culture was applied onto an YM plate of 2% glucose containing 8% (v/v) ethanol for ethanol tolerance test using 10-fold serial dilutions of cell suspension. The culture plates were incubated at 30°C and examined 4 days after incubation. Tolerance to inhibitors furfural and HMF were examined in a similar manner on YM plates of 2% glucose containing 10 mM each of furfural and HMF 7 days after incubation.

Cell viability was examined for cultures grown under a challenge with 8% of ethanol over time. The time point after 6-h pre-culture when ethanol was added into the culture was designated as 0 h. Samples were taken starting at 24 h after the ethanol challenge until 168 h with a 24-h interval. Cell growth was examined on a solid YM using an assay similar as described above.

### Sample collection and HPLC analysis

Cell growth was monitored by absorbance at OD_600 _under ethanol stress. Samples were taken and cells harvested at 0, 1, 6, 24, and 48 h after the 8% ethanol addition for mRNA expression analysis using procedures as previous described [41]. Yeast cells were immediately frozen on dry ice and then stored at -80°C until use. Samples of culture supernatants were taken periodically from 0 h to 120 h after the ethanol challenge for metabolic profiling analysis. Glucose consumption, ethanol conversion, acetic acid, and glycerol production were measured using an HPLC system composed of a Waters 717 plus autosampler controlled at 10°C, Waters 590 programmable pump, a Fast Acid column (Bio-Rad Laboratories, Hercules, CA) proceeded by a Microguard Cation H guard cartridge, a Spectra-Physics Spectra 100 variable wavelength UV detector (215 nm), and a Waters 2414 refractive index detector. The column was maintained at 65°C, and samples were eluted with 1.6 mM H_2_SO_4 _at 0.6 ml/min. A standard curve was constructed for each detected chemical and metabolic conversion product for HPLC assays as described previously [33,38].

### Pathway-based qRT-PCR array assays

Pathway-based qRT-PCR array assays were carried out using 96-well plates. Based on microarray studies, 175 genes involved in ethanol tolerance and ethanol production were selected for quantitative transcription analysis using qRT-PCR arrays. A recently developed robust data acquisition reference *CAB *[40] and mRNA calibration standard [41] were applied for the qRT-PCR arrays. Primers of selected genes were designed (Additional File 4) using Primer 3 [72] with manual editing based on sequences of the Saccharomyces Genome Database [73]. Gene-specific amplification was verified by PCR and dissociation curve analysis. The length of designed amplicons of most tested genes ranged from 100 to 150 bp with a few exceptions of shorter amplicons down to 75 bp and one longer up to 210 bp.

Total RNA was isolated from each of two biological and two technical replications using procedures as previously described [41,74]. RNA integrity was verified by gel electrophoresis and NanoDrop Spectrophotometer ND-100 (NanoDrop Technologies, Inc., Wilmington, DE). Reverse transcription reactions applying the robust mRNA controls were carried out using procedures as previously described [40]. SYBR Green iTaq PCR master mix (BioRad Laboratories) was applied for each qRT-PCR reaction. For each reaction, a total of 25 μl was used consisting of 12.5 μl 2X SYBR Green MasterMix, 0.5 μl each of forward and reverse primer (10 μM each), 0.25 μl cDNA template, and 11.25 μl H_2_O. On each 96-well plate, reactions of qRT-PCR were carried out with two replications for each control gene except for the control *CAB *of three replications. All reactions of the tested target gene were run in duplicate. Control gene *B2M *served as a non template negative control for each plate. PCR was run on an ABI 7500 real time PCR system using a defined profile as previously described [40]. A total of 80 96-well plates were applied for the qRT-PCR array assays. Transcription copy number of target genes was estimated using an equation based on the standard mRNA reference and master equation [40,75] as follows:(2)

where mRNA is an estimated value in pg using the master equation and Amplicon is the amplified bp-length of an interested target gene.

### Data analysis

Mean values of three *CAB *amplifications on a plate were designated and used as a constant reference to set up a manual threshold at 26 Ct (cycle number) for data analysis. This sole reference served as a constant standard for data acquisition and analysis for each and every qRT-PCR run. MasterqRT-PCR C^++ ^program http://cs1.bradley.edu/~nri/MasterqRT-PCR/[40] was used to generate a master equation, evaluate PCR amplification efficiency, and estimate transcript copy numbers as described previously [37,40]. Additional statistical analyses were performed using statistical function tools of Microsoft Excel. Quantitative expression data were correlated to metabolic profiling for ethanol tolerant strain Y-50316 and its parental strain Y-50049. Standard Gene Ontology (GO) annotations were carried out using GO Slim Mapper http://www.yeastgenome.org/cgi-bin/GO/goSlimMapper.pl. DNA binding motifs of transcription factors were annotated for candidate and key genes for ethanol tolerance and subsequent ethanol fermentation using YEASTRACT [76]. Previous knowledge of KEGG pathway database http://www.genome.jp/kegg/kegg.html was referenced for pathway constructions.

## Authors' contributions

ZLL designed the qRT-PCR array and conceived the experiment. MM performed strain adaptation, experimental fermentation, sample collection, RNA extraction, qRT-PCR and data analysis. ZLL and MM analyzed the data and wrote the manuscript. All authors read and approved the final manuscript.

## Supplementary Material

Additional file 1 Performance of standard curves derived from robust universal standard controls using *CAB *as the sole reference to set Ct at 26 by manual as threshold for data acquisition over 80 individual plate reactions on Applied Biosystems 7500 real time PCR System applying MasterqRT-PCR C^++ ^program http://cs1.bradley.edu/~nri/MasterqRT-PCR/ Click here for file

Additional file 2 **Mean estimate of mRNA abundance in forms of transcript copy numbers (n × 10^7^) for selected genes of *Saccharomyces cerevisiae *NRRL Y-50316 and NRRL Y-50049 in response to ethanol challenge over a time-course study**. Click here for file

Additional file 3 **Gene Ontology (GO) categories and terms of candidate and key genes for ethanol tolerance and fermentation under stress in *Saccharomyces cerevisiae***. Click here for file

Additional file 4 **Primers used for mRNA expression analysis by real-time qRT-PCR using SYBR Green**. Click here for file
