# Supplementary material for: Quantitative transcription dynamic analysis reveals candidate genes and key regulators for ethanol tolerance in Saccharomyces cerevisiae
Source: BMC Microbiol. 2010 Jun 10;10:169. doi: 10.1186/1471-2180-10-169 (PMC2903563; doi:10.1186/1471-2180-10-169)
Supplement: Additional file 1 — Performance of standard curves derived from robust universal standard controls using CAB as the sole reference to set Ct at 26 by manual as threshold for data acquisition over 80 individual plate reactions on Applied Biosystems 7500 real time PCR System applying MasterqRT-PCR C++ program http://cs1.bradley.edu/~nri/MasterqRT-PCR/ [file 1471-2180-10-169-S1.DOC]

Additional File 1. Performance of standard curves derived from robust universal standard controls using *CAB* as the sole reference to set Ct at 26 by manual as threshold for data acquisition over 80 individual plate reactions on Applied Biosystems 7500 real time PCR System applying MasterqRT-PCR C^++^ program (http://cs1.bradley.edu/~nri/MasterqRT-PCR/)

| Plate | Slope | Intercept | R^2^ |
| --- | --- | --- | --- |
| 1 | -3.3297 | 25.689 | 0.9979 |
| 2 | -3.3049 | 25.5745 | 0.9969 |
| 3 | -3.4499 | 25.8378 | 0.9997 |
| 4 | -3.4276 | 25.8128 | 0.9995 |
| 5 | -3.5083 | 25.847 | 0.9998 |
| 6 | -3.4923 | 25.8784 | 0.9998 |
| 7 | -3.4537 | 25.6703 | 0.9989 |
| 8 | -3.4724 | 25.7439 | 0.9993 |
| 9 | -3.4609 | 25.8998 | 0.9998 |
| 10 | -3.4421 | 25.8562 | 0.9998 |
| 11 | -3.4386 | 25.8914 | 0.9999 |
| 12 | -3.4331 | 25.8415 | 0.9997 |
| 13 | -3.4876 | 26.0228 | 1 |
| 14 | -3.4643 | 25.979 | 1 |
| 15 | -3.506 | 26.042 | 0.9998 |
| 16 | -3.495 | 25.97 | 0.9999 |
| 17 | -3.459 | 26.0213 | 0.9998 |
| 18 | -3.4863 | 26.0997 | 0.9997 |
| 19 | -3.499 | 25.988 | 0.9999 |
| 20 | -3.5141 | 26.0802 | 0.9998 |
| 21 | -3.4956 | 26.1054 | 0.9997 |
| 22 | -3.4939 | 26.1058 | 0.9994 |
| 23 | -3.5207 | 26.0943 | 0.9995 |
| 24 | -3.497 | 26.0173 | 0.9998 |
| 25 | -3.5089 | 25.8191 | 0.9982 |
| 26 | -3.5207 | 25.8176 | 0.9977 |
| 27 | -3.5261 | 26.0042 | 0.9996 |
| 28 | -3.4901 | 25.8622 | 0.9997 |
| 29 | -3.4743 | 25.9457 | 0.9994 |
| 30 | -3.4851 | 26.0489 | 0.9994 |
| 31 | -3.5263 | 25.9897 | 0.9997 |
| 32 | -3.5109 | 25.9398 | 0.9997 |
| 33 | -3.5449 | 25.9945 | 0.9992 |
| 34 | -3.5167 | 25.9296 | 0.9994 |
| 35 | -3.5263 | 25.9497 | 0.9994 |
| 36 | -3.533 | 25.9627 | 0.9996 |
| 37 | -3.498 | 25.9693 | 1 |
| 38 | -3.4887 | 25.9636 | 1 |
| 39 | -3.4937 | 25.997 | 0.9999 |
| 40 | -3.513 | 26.0527 | 0.9998 |
| 41 | -3.4187 | 25.9836 | 0.9991 |
| 42 | -3.4559 | 25.9331 | 0.9997 |
| 43 | -3.491 | 25.9753 | 0.9995 |
| 44 | -3.4734 | 25.9859 | 0.9997 |
| 45 | -3.399 | 26.1013 | 0.9985 |
| 46 | -3.3974 | 26.0806 | 0.9987 |
| 47 | -3.4377 | 25.9516 | 0.9999 |
| 48 | -3.4466 | 26.0041 | 0.9998 |
| 49 | -3.4194 | 25.8879 | 0.9996 |
| 50 | -3.4144 | 25.8746 | 0.9996 |
| 51 | -3.413 | 25.866 | 0.9996 |
| 52 | -3.3849 | 25.8645 | 0.9996 |
| 53 | -3.4143 | 25.899 | 0.9998 |
| 54 | -3.4331 | 25.8749 | 0.9998 |
| 55 | -3.4204 | 25.9232 | 0.9999 |
| 56 | -3.4261 | 25.9342 | 1 |
| 57 | -3.4449 | 25.9578 | 0.9995 |
| 58 | -3.446 | 25.9753 | 0.9993 |
| 59 | -3.4391 | 25.9135 | 0.9995 |
| 60 | -3.4501 | 25.9355 | 0.9995 |
| 61 | -3.4974 | 26.0806 | 0.9999 |
| 62 | -3.472 | 26.0707 | 0.9998 |
| 63 | -3.4584 | 25.9259 | 0.9998 |
| 64 | -3.4383 | 25.907 | 0.9998 |
| 65 | -3.4436 | 25.9414 | 0.9999 |
| 66 | -3.4419 | 25.9351 | 0.9999 |
| 67 | -3.4546 | 25.9801 | 0.9996 |
| 68 | -3.4407 | 25.9543 | 0.9998 |
| 69 | -3.4566 | 25.9208 | 0.9999 |
| 70 | -3.4674 | 25.9839 | 0.9999 |
| 71 | -3.473 | 25.9293 | 1 |
| 72 | -3.4637 | 25.9136 | 1 |
| 73 | -3.44 | 25.9333 | 0.9998 |
| 74 | -3.447 | 25.974 | 0.9998 |
| 75 | -3.4559 | 25.9665 | 0.9998 |
| 76 | -3.4441 | 25.9202 | 0.9999 |
| 77 | -3.3983 | 25.967 | 1 |
| 78 | -3.3789 | 25.9758 | 1 |
| 79 | -3.3744 | 25.8679 | 0.9999 |
| 80 | -3.3864 | 25.9019 | 0.9999 |
| Mean | -3.4593 | 25.9411 | 0.9996 |
| stdev | 0.0458 | 0.0966 | 0.0005 |
