# Supplementary material for: Quantitative transcription dynamic analysis reveals candidate genes and key regulators for ethanol tolerance in Saccharomyces cerevisiae
Source: BMC Microbiol. 2010 Jun 10;10:169. doi: 10.1186/1471-2180-10-169 (PMC2903563; doi:10.1186/1471-2180-10-169)
Supplement: Additional file 2 — Mean estimate of mRNA abundance in forms of transcript copy numbers (n × 107) for selected genes of Saccharomyces cerevisiae NRRL Y-50316 and NRRL Y-50049 in response to ethanol challenge over a time-course study. [file 1471-2180-10-169-S2.DOC]

Additional File 2. Mean estimate of mRNA abundance in forms of transcript copy numbers (n×10^7^) for selected genes of *Saccharomyces cerevisiae* NRRL Y-50316 and NRRL Y-50049 in response to ethanol challenge over a time-course study

| Gene/ORF | Category | Y-50316 | | | | | | | | | | Y-50049 | | | | | | | | | |
| --- | --- | --- | --- | --- | --- | --- | --- | --- | --- | --- | --- | --- | --- | --- | --- | --- | --- | --- | --- | --- | --- |
|  |  | 0 h | | 1 h | | 6 h | | 24 h | | 48h | | 0 h | | 1 h | | 6 h | | 24 h | | 48 h | |
|  |  | Mean | Stdev | Mean | Stdev | Mean | Stdev | Mean | Stdev | Mean | Stdev | Mean | Stdev | Mean | Stdev | Mean | Stdev | Mean | Stdev | Mean | Stdev |
| *ACC1*/YNR016C | Fatty  Acid  Metabolism | 40.71 | 0.84 | 11.54 | 1.03 | 9.65 | 0.67 | 9.84 | 0.65 | 6.39 | 2.01 | 57.47 | 2.16 | 11.28 | 0.75 | 8.71 | 0.49 | 4.64 | 0.17 | 3.33 | 0.33 |
| *FAS1*/YKL182W |  | 127.01 | 3.44 | 47.61 | 6.40 | 54.79 | 6.19 | 58.43 | 9.86 | 35.88 | 8.98 | 129.39 | 13.02 | 32.39 | 16.62 | 44.27 | 14.65 | 26.37 | 1.05 | 16.27 | 1.13 |
| *FAS2*/YPL231W |  | 62.94 | 1.29 | 20.81 | 1.19 | 27.94 | 0.24 | 30.07 | 1.04 | 26.61 | 7.38 | 72.05 | 2.81 | 17.16 | 1.69 | 19.99 | 3.44 | 9.80 | 0.74 | 4.09 | 0.52 |
| *ELO1*/YJL196C |  | 27.89 | 2.36 | 13.58 | 2.59 | 22.69 | 4.38 | 30.51 | 1.53 | 17.53 | 3.58 | 17.23 | 0.28 | 8.49 | 2.69 | 12.46 | 3.11 | 6.67 | 0.27 | 4.58 | 0.18 |
| *FEN1*/YCR034W |  | 86.74 | 1.24 | 22.13 | 0.67 | 35.98 | 3.66 | 55.91 | 1.25 | 18.20 | 4.59 | 87.12 | 9.55 | 24.83 | 8.00 | 49.59 | 14.54 | 24.27 | 1.31 | 19.91 | 1.19 |
| *SUR4*/YLR372W |  | 107.03 | 2.77 | 27.18 | 1.95 | 44.18 | 5.41 | 70.61 | 2.31 | 28.84 | 5.73 | 126.37 | 8.34 | 30.79 | 8.43 | 99.00 | 28.50 | 48.06 | 3.23 | 27.07 | 0.55 |
| *OLE1*/YGL055W |  | 160.86 | 8.12 | 35.07 | 2.31 | 81.42 | 8.43 | 64.28 | 18.00 | 48.70 | 10.92 | 250.86 | 39.29 | 34.61 | 14.33 | 27.93 | 7.44 | 35.46 | 2.32 | 14.49 | 0.44 |
| *IFA38*/YBR159W |  | 49.42 | 2.84 | 25.86 | 4.06 | 34.42 | 6.55 | 44.23 | 2.85 | 24.23 | 4.92 | 49.05 | 8.69 | 22.26 | 6.11 | 41.94 | 14.46 | 17.79 | 0.87 | 13.02 | 0.88 |
| *PHS1*/YJL097W |  | 41.51 | 0.92 | 17.29 | 0.96 | 31.67 | 5.38 | 36.82 | 0.83 | 23.80 | 4.67 | 34.00 | 3.35 | 13.46 | 1.83 | 35.15 | 9.81 | 19.00 | 1.48 | 14.23 | 0.72 |
| *TSC13*/YDL015C |  | 38.41 | 5.99 | 24.73 | 4.93 | 38.97 | 12.31 | 57.69 | 7.69 | 32.23 | 4.40 | 42.86 | 1.90 | 35.34 | 16.08 | 54.60 | 25.71 | 19.27 | 1.89 | 12.87 | 0.60 |
| *HFA1*/YMR207C |  | 9.33 | 2.41 | 6.35 | 1.31 | 4.66 | 1.28 | 4.21 | 0.61 | 4.03 | 1.49 | 10.37 | 1.23 | 7.71 | 0.20 | 4.14 | 0.56 | 3.03 | 0.31 | 3.32 | 0.58 |
| *MCT1*/YOR221C |  | 12.55 | 0.96 | 5.78 | 0.90 | 7.97 | 3.50 | 6.41 | 0.54 | 5.25 | 1.38 | 16.67 | 3.10 | 10.92 | 2.73 | 16.22 | 8.04 | 6.34 | 0.44 | 5.75 | 0.13 |
| *CEM1*/YER061C |  | 18.34 | 3.54 | 10.62 | 1.54 | 25.86 | 5.17 | 31.73 | 2.09 | 23.18 | 4.43 | 49.02 | 6.81 | 16.62 | 3.38 | 51.25 | 15.41 | 21.87 | 0.98 | 19.95 | 0.19 |
| *OAR1*/YKL055C |  | 11.10 | 2.80 | 7.96 | 1.40 | 7.84 | 2.79 | 9.37 | 0.25 | 8.09 | 2.28 | 9.01 | 0.76 | 14.29 | 2.44 | 6.98 | 1.80 | 4.69 | 0.18 | 4.15 | 0.29 |
| *HTD2*/YHR067W |  | 8.00 | 2.32 | 6.50 | 1.28 | 7.86 | 2.23 | 7.88 | 0.19 | 6.91 | 1.82 | 7.21 | 0.90 | 5.31 | 0.35 | 8.09 | 2.49 | 3.85 | 0.17 | 3.54 | 0.13 |
| *ETR1*/YBR026C |  | 14.01 | 2.70 | 14.75 | 2.56 | 23.64 | 4.85 | 31.92 | 1.20 | 26.22 | 3.53 | 15.36 | 1.61 | 24.81 | 3.20 | 19.86 | 4.57 | 10.47 | 0.18 | 7.99 | 0.39 |
| *ERG10*/YPL028W | Ergosterol  Metabolism | 154.56 | 5.66 | 84.04 | 9.88 | 106.29 | 14.63 | 84.46 | 8.22 | 73.45 | 11.12 | 162.53 | 7.70 | 52.66 | 15.18 | 106.07 | 31.87 | 53.27 | 1.34 | 26.93 | 0.74 |
| *ERG13*/YML126C |  | 186.00 | 10.54 | 53.42 | 8.12 | 119.02 | 14.63 | 120.96 | 16.26 | 81.43 | 16.46 | 245.89 | 11.33 | 79.91 | 26.60 | 195.72 | 59.18 | 117.21 | 3.71 | 71.15 | 2.25 |
| *HMG1*/YML075C |  | 65.31 | 12.69 | 31.33 | 6.25 | 65.83 | 8.07 | 69.67 | 18.57 | 37.22 | 8.07 | 101.62 | 4.02 | 24.90 | 10.63 | 57.54 | 19.65 | 34.72 | 2.27 | 21.12 | 0.63 |
| *HMG2*/YLR450W |  | 16.45 | 0.86 | 7.93 | 1.39 | 8.93 | 1.42 | 12.58 | 1.23 | 8.57 | 1.87 | 14.14 | 1.16 | 5.08 | 0.96 | 12.09 | 3.42 | 4.79 | 0.06 | 2.95 | 0.11 |
| *ERG12*/YMR208W |  | 50.71 | 4.60 | 24.22 | 3.09 | 33.95 | 6.66 | 34.75 | 4.42 | 28.22 | 5.50 | 62.16 | 1.66 | 19.79 | 5.75 | 50.71 | 14.80 | 26.99 | 1.73 | 18.76 | 0.40 |
| *ERG8*/YMR220W |  | 37.16 | 4.59 | 16.35 | 2.47 | 26.83 | 4.91 | 31.30 | 2.48 | 22.03 | 3.95 | 38.77 | 3.62 | 17.31 | 4.01 | 31.78 | 7.43 | 17.56 | 0.24 | 9.66 | 0.83 |
| *MVD1*/YNR043W |  | 38.31 | 4.64 | 13.61 | 2.41 | 25.87 | 7.44 | 24.93 | 4.08 | 21.62 | 5.60 | 50.03 | 2.80 | 16.41 | 5.92 | 49.04 | 18.94 | 26.21 | 0.82 | 18.83 | 0.95 |
| *ERG20*/YJL167W |  | 111.61 | 12.70 | 96.62 | 12.76 | 116.65 | 21.12 | 119.78 | 16.65 | 77.06 | 15.58 | 130.48 | 7.61 | 76.61 | 24.43 | 168.99 | 53.19 | 80.21 | 3.27 | 54.14 | 1.37 |
| *ERG9*/YHR190W |  | 48.43 | 6.34 | 34.85 | 4.95 | 54.14 | 14.21 | 44.65 | 4.30 | 41.08 | 8.18 | 62.81 | 6.08 | 38.83 | 8.92 | 78.46 | 26.52 | 34.45 | 1.00 | 20.09 | 0.40 |
| *ERG1*/YGR175C |  | 135.97 | 10.30 | 35.68 | 3.11 | 63.75 | 10.48 | 86.96 | 9.42 | 48.30 | 9.75 | 255.55 | 11.97 | 51.23 | 22.45 | 107.14 | 31.50 | 49.78 | 2.41 | 22.62 | 1.03 |
| *ERG7*/YHR072W |  | 34.60 | 4.40 | 8.25 | 0.80 | 14.62 | 1.97 | 15.46 | 0.73 | 7.08 | 2.04 | 37.38 | 0.57 | 8.79 | 2.28 | 21.12 | 6.35 | 7.73 | 0.51 | 3.97 | 0.13 |
| *ERG11*/YHR007C |  | 173.46 | 13.94 | 50.04 | 1.85 | 101.49 | 23.49 | 107.20 | 19.02 | 97.45 | 14.38 | 240.23 | 5.91 | 81.11 | 34.08 | 141.75 | 38.51 | 98.76 | 6.36 | 92.72 | 6.26 |
| *ERG24*/YNL280C |  | 46.71 | 2.19 | 15.13 | 1.79 | 41.70 | 5.14 | 48.54 | 4.18 | 34.77 | 4.10 | 61.43 | 4.34 | 16.26 | 4.72 | 45.48 | 12.12 | 23.07 | 1.20 | 15.41 | 0.05 |
| *ERG25*/YGR060W |  | 314.79 | 4.62 | 82.75 | 1.82 | 222.28 | 32.71 | 163.40 | 21.67 | 148.80 | 19.18 | 376.18 | 7.94 | 98.70 | 31.02 | 204.33 | 57.41 | 130.12 | 3.04 | 63.40 | 0.73 |
| *ERG26*/YGL001C |  | 78.06 | 4.17 | 30.67 | 3.15 | 71.45 | 12.94 | 61.81 | 4.74 | 61.26 | 6.97 | 80.20 | 4.89 | 28.24 | 9.51 | 64.33 | 19.02 | 38.13 | 0.81 | 28.13 | 1.00 |
| *ERG27*/YLR100W |  | 50.85 | 4.91 | 20.13 | 0.67 | 35.41 | 6.88 | 22.15 | 1.87 | 16.25 | 2.95 | 74.85 | 5.91 | 27.88 | 9.07 | 60.57 | 20.56 | 32.29 | 0.93 | 20.41 | 0.80 |
| *ERG6*/YML008C |  | 120.49 | 8.75 | 26.29 | 2.29 | 66.53 | 12.64 | 39.33 | 3.00 | 25.88 | 5.09 | 152.49 | 8.75 | 37.55 | 13.27 | 61.72 | 19.00 | 33.07 | 2.50 | 18.59 | 0.39 |
| *ERG2*/YMR202W |  | 229.78 | 5.73 | 57.68 | 4.13 | 121.43 | 22.57 | 49.43 | 7.10 | 32.98 | 6.36 | 330.14 | 20.93 | 81.26 | 37.27 | 180.66 | 63.87 | 106.50 | 0.82 | 94.13 | 5.71 |
| *ERG3*/YLR056W |  | 23.91 | 4.45 | 4.48 | 0.55 | 16.20 | 6.19 | 8.80 | 2.81 | 9.04 | 2.34 | 32.79 | 5.17 | 6.52 | 3.86 | 22.94 | 12.65 | 10.75 | 0.36 | 9.03 | 0.71 |
| *ERG5*/YMR015C |  | 39.29 | 7.38 | 15.97 | 2.78 | 46.79 | 13.39 | 44.71 | 12.61 | 28.69 | 7.54 | 63.92 | 3.00 | 14.56 | 6.68 | 47.39 | 23.06 | 17.96 | 1.92 | 8.53 | 0.56 |
| *ERG4*/YGL012W |  | 73.98 | 1.59 | 23.08 | 1.88 | 27.90 | 5.52 | 21.86 | 1.09 | 14.81 | 2.50 | 47.97 | 4.13 | 32.74 | 7.83 | 17.01 | 5.08 | 10.72 | 0.25 | 7.89 | 0.31 |
| *ERG28*/YER044C |  | 132.80 | 9.12 | 80.31 | 7.79 | 114.38 | 15.76 | 139.88 | 16.69 | 95.65 | 10.68 | 290.16 | 25.18 | 121.38 | 45.21 | 204.54 | 66.20 | 110.04 | 1.91 | 101.25 | 9.59 |
| *ERG29*/YMR134W |  | 31.08 | 0.78 | 11.91 | 1.79 | 22.40 | 5.76 | 22.26 | 2.72 | 15.26 | 3.40 | 41.81 | 3.18 | 19.01 | 6.06 | 29.35 | 7.13 | 20.72 | 1.64 | 19.28 | 1.42 |
| *PRO1*/YDR300C | Proline  Metabolism | 30.83 | 2.31 | 19.10 | 2.06 | 13.15 | 2.90 | 17.04 | 0.35 | 13.29 | 2.56 | 18.76 | 1.60 | 13.19 | 1.93 | 17.80 | 3.36 | 8.81 | 0.37 | 5.29 | 0.23 |
| *PRO2*/YOR323C |  | 42.51 | 0.81 | 17.62 | 1.74 | 25.91 | 6.04 | 29.93 | 0.86 | 18.06 | 2.58 | 52.09 | 6.14 | 16.07 | 5.09 | 48.93 | 14.20 | 21.03 | 0.73 | 13.51 | 0.36 |
| *PRO3*/YER023W |  | 78.89 | 2.44 | 31.76 | 3.82 | 48.83 | 9.12 | 59.15 | 1.31 | 32.80 | 6.02 | 102.94 | 11.98 | 39.95 | 11.23 | 65.03 | 18.13 | 31.86 | 1.30 | 20.42 | 0.18 |
| *PUT1*/YLR142W |  | 4.90 | 1.82 | 6.68 | 1.17 | 23.77 | 3.59 | 16.06 | 0.75 | 43.15 | 5.40 | 8.79 | 1.09 | 44.98 | 6.34 | 33.70 | 5.87 | 52.75 | 1.55 | 22.49 | 0.33 |
| *PUT2*/YHR037W |  | 2.27 | 0.62 | 2.71 | 0.77 | 3.67 | 0.75 | 4.36 | 0.39 | 4.34 | 0.66 | 2.85 | 0.48 | 4.85 | 1.70 | 4.62 | 1.38 | 2.30 | 0.27 | 1.18 | 0.09 |
| *NTH1*/YDR001C | Trehalose  and  Glycogen  Mebabolism | 18.05 | 1.91 | 26.91 | 3.61 | 47.51 | 9.40 | 55.87 | 2.25 | 52.90 | 5.64 | 21.03 | 1.98 | 13.44 | 2.74 | 42.84 | 8.87 | 24.24 | 1.22 | 11.08 | 0.20 |
| *NTH2*/YBR001C |  | 15.81 | 2.99 | 21.49 | 3.44 | 32.57 | 5.12 | 42.20 | 1.46 | 42.72 | 6.08 | 15.29 | 1.46 | 13.36 | 0.33 | 21.89 | 5.55 | 13.13 | 0.58 | 7.83 | 1.04 |
| *ATH1*/YPR026W |  | 10.13 | 2.87 | 14.52 | 2.39 | 19.84 | 3.48 | 20.16 | 1.06 | 19.15 | 3.69 | 9.37 | 0.83 | 16.13 | 1.35 | 11.13 | 2.12 | 5.51 | 0.27 | 3.75 | 0.37 |
| *TPS2*/YDR074W |  | 3.53 | 0.59 | 6.72 | 1.42 | 8.54 | 0.76 | 11.54 | 0.63 | 5.52 | 2.15 | 6.74 | 0.66 | 21.80 | 4.53 | 7.96 | 2.37 | 3.54 | 0.45 | 1.45 | 0.05 |
| *TPS1*/YBR126C |  | 59.58 | 4.80 | 146.70 | 20.52 | 189.47 | 38.95 | 185.10 | 15.85 | 105.72 | 15.77 | 97.79 | 7.11 | 130.58 | 28.57 | 161.87 | 48.65 | 68.77 | 7.45 | 41.72 | 4.29 |
| *TPS3*/YMR261C |  | 31.21 | 1.63 | 34.35 | 5.68 | 45.07 | 10.51 | 53.85 | 2.61 | 40.72 | 6.33 | 47.79 | 4.23 | 39.98 | 8.98 | 58.94 | 17.19 | 26.24 | 1.11 | 12.10 | 1.62 |
| *TSL1*/YML100W |  | 27.67 | 1.54 | 151.75 | 16.44 | 163.94 | 18.85 | 145.71 | 10.25 | 108.11 | 17.19 | 47.03 | 3.94 | 85.03 | 13.19 | 105.90 | 22.54 | 52.73 | 1.83 | 19.59 | 1.41 |
| *UGP1*/YKL035W |  | 105.79 | 3.54 | 232.50 | 46.77 | 146.23 | 43.67 | 182.70 | 15.49 | 112.47 | 14.69 | 96.16 | 8.78 | 244.79 | 65.28 | 148.24 | 56.78 | 54.95 | 4.67 | 32.02 | 0.89 |
| *GPH1*/YPR160W |  | 15.48 | 3.62 | 83.23 | 18.06 | 231.14 | 74.39 | 320.35 | 14.35 | 285.93 | 57.17 | 16.14 | 2.77 | 39.10 | 6.58 | 106.53 | 38.41 | 72.56 | 0.79 | 56.85 | 2.60 |
| *GSY1/YFR015C* |  | 10.92 | 2.73 | 65.74 | 9.28 | 43.24 | 8.72 | 39.25 | 1.16 | 19.98 | 4.22 | 19.56 | 1.07 | 30.52 | 4.90 | 48.00 | 12.99 | 20.78 | 0.21 | 8.76 | 0.07 |
| *GSY2*/YLR258W |  | 7.35 | 1.94 | 16.45 | 3.43 | 42.76 | 6.62 | 43.25 | 1.49 | 32.15 | 5.99 | 13.43 | 1.87 | 18.27 | 1.39 | 27.52 | 5.91 | 19.51 | 0.20 | 8.11 | 0.28 |
| *TRP2*/YER090W | Tryptophan  Metabolism | 42.35 | 0.51 | 14.45 | 1.65 | 30.05 | 8.10 | 51.40 | 4.07 | 31.09 | 5.94 | 54.05 | 6.81 | 46.13 | 14.28 | 52.54 | 17.01 | 33.50 | 0.71 | 23.42 | 1.52 |
| *TRP3*/YKL211C |  | 28.97 | 2.54 | 15.41 | 3.86 | 14.76 | 3.33 | 20.62 | 3.51 | 15.05 | 5.14 | 23.29 | 5.06 | 14.15 | 4.73 | 29.61 | 13.13 | 12.85 | 0.35 | 6.50 | 0.47 |
| *TRP4*/YDR354W |  | 28.18 | 0.58 | 15.46 | 2.55 | 15.40 | 5.23 | 20.17 | 1.40 | 10.87 | 2.54 | 24.22 | 3.97 | 16.65 | 4.35 | 19.65 | 7.47 | 10.14 | 0.68 | 8.21 | 0.26 |
| *TRP1*/YDR007W |  | 21.68 | 1.85 | 15.05 | 1.61 | 11.52 | 2.52 | 14.15 | 0.52 | 9.91 | 1.78 | 23.12 | 4.29 | 12.44 | 2.06 | 14.40 | 4.30 | 8.43 | 0.14 | 7.77 | 0.20 |
| *TRP5*/YGL026C |  | 65.92 | 1.50 | 22.94 | 3.66 | 45.29 | 16.01 | 62.24 | 6.71 | 32.77 | 6.51 | 44.69 | 2.12 | 16.84 | 5.15 | 57.28 | 22.16 | 22.27 | 1.55 | 10.36 | 0.26 |
| *GPD1*/YDL022W | Glycerol  Metabolism | 68.10 | 3.91 | 40.55 | 4.22 | 49.54 | 6.18 | 59.56 | 1.85 | 25.65 | 4.76 | 52.09 | 11.77 | 72.25 | 21.59 | 54.09 | 20.52 | 16.99 | 1.74 | 7.81 | 0.09 |
| *GPD2*/YOL059W |  | 36.65 | 0.98 | 54.78 | 6.88 | 24.68 | 5.57 | 26.14 | 1.78 | 24.03 | 4.38 | 47.35 | 8.42 | 87.88 | 26.26 | 51.30 | 14.76 | 25.66 | 0.44 | 16.73 | 0.35 |
| *HOR2*/YER062C |  | 73.28 | 1.74 | 51.66 | 6.63 | 39.60 | 5.38 | 53.74 | 4.63 | 24.78 | 3.47 | 115.49 | 9.29 | 62.21 | 19.36 | 62.14 | 19.82 | 30.56 | 1.59 | 20.96 | 0.29 |
| *RHR2/*YIL053W |  | 289.40 | 16.66 | 199.86 | 23.83 | 72.66 | 9.87 | 72.81 | 3.40 | 25.03 | 5.20 | 250.53 | 33.45 | 243.36 | 59.19 | 130.78 | 27.84 | 55.86 | 1.83 | 33.86 | 1.44 |
| *GUT1*/YHL032C |  | 15.06 | 1.53 | 13.91 | 2.99 | 18.19 | 4.31 | 18.04 | 0.36 | 11.31 | 2.27 | 14.29 | 2.09 | 15.06 | 2.95 | 18.28 | 5.23 | 9.48 | 0.48 | 6.76 | 0.08 |
| *GUT2*/YIL155C |  | 5.30 | 1.43 | 7.90 | 1.48 | 10.50 | 2.91 | 6.84 | 0.18 | 6.58 | 1.62 | 7.33 | 0.69 | 21.48 | 3.49 | 8.02 | 1.27 | 5.38 | 0.30 | 3.34 | 0.30 |
| *GCY1*/YOR120W |  | 32.32 | 4.00 | 27.09 | 4.96 | 126.33 | 24.52 | 159.92 | 6.06 | 140.78 | 15.43 | 29.43 | 2.87 | 31.74 | 5.76 | 120.56 | 35.80 | 64.55 | 1.47 | 48.65 | 1.43 |
| *DAK1*/YML070W |  | 37.13 | 3.25 | 68.83 | 11.60 | 62.57 | 11.94 | 58.75 | 2.64 | 56.31 | 7.45 | 31.22 | 2.54 | 49.20 | 8.56 | 62.41 | 14.11 | 20.15 | 0.74 | 8.41 | 0.31 |
| *PFS1*/YHR185C |  | 0.232 | 0.128 | 0.249 | 0.100 | 0.194 | 0.093 | 0.179 | 0.008 | 0.165 | 0.075 | 0.333 | 0.019 | 0.299 | 0.053 | 0.136 | 0.031 | 0.112 | 0.019 | 0.140 | 0.004 |
| *GUP1*/YGL084C |  | 21.45 | 2.08 | 18.09 | 1.74 | 15.80 | 1.73 | 21.06 | 0.65 | 14.87 | 2.62 | 18.21 | 1.57 | 11.25 | 2.02 | 18.23 | 5.41 | 9.04 | 0.18 | 6.01 | 0.47 |
| *GUP2*/YPL189W |  | 11.43 | 1.45 | 4.74 | 0.65 | 3.62 | 1.01 | 6.41 | 0.19 | 3.84 | 1.22 | 6.26 | 0.92 | 4.53 | 0.12 | 6.11 | 1.61 | 3.69 | 0.20 | 3.11 | 0.22 |
| *HSP10*/YOR020C | Heat  Shock  Protein  Family | 155.86 | 12.53 | 209.93 | 14.65 | 131.99 | 32.25 | 132.59 | 4.76 | 131.66 | 19.36 | 166.45 | 10.73 | 173.89 | 41.78 | 233.81 | 67.09 | 117.07 | 5.43 | 126.76 | 4.48 |
| *HSP12*/YFL014W |  | 66.52 | 7.99 | 503.37 | 53.64 | 751.12 | 184.77 | 645.90 | 21.99 | 535.72 | 69.86 | 96.20 | 6.15 | 409.06 | 23.85 | 202.62 | 25.44 | 127.47 | 2.67 | 114.45 | 4.29 |
| *HSP26*/YBR072W |  | 17.55 | 2.63 | 1068.91 | 96.56 | 581.60 | 92.18 | 613.19 | 29.04 | 1052.63 | 77.70 | 19.37 | 1.36 | 1152.18 | 222.31 | 673.78 | 160.86 | 344.51 | 3.23 | 295.68 | 8.37 |
| *HSP30*/YCR021C |  | 9.18 | 4.58 | 73.01 | 12.12 | 31.94 | 4.46 | 68.30 | 9.28 | 230.50 | 4.55 | 9.65 | 0.68 | 471.24 | 119.40 | 44.45 | 10.13 | 31.20 | 1.27 | 28.61 | 1.05 |
| *HSP31*/YDR533C |  | 92.50 | 3.28 | 160.24 | 28.30 | 355.83 | 84.26 | 458.85 | 25.70 | 418.85 | 54.85 | 44.85 | 3.38 | 57.41 | 13.97 | 246.04 | 66.68 | 93.73 | 3.06 | 78.99 | 3.36 |
| *HSP32*/YPL280W |  | 9.74 | 3.60 | 13.34 | 3.34 | 30.50 | 7.56 | 27.36 | 3.04 | 29.84 | 7.09 | 12.87 | 2.16 | 19.88 | 2.59 | 27.10 | 7.95 | 17.46 | 0.09 | 12.92 | 1.15 |
| *HSP40*/YNL064C |  | 188.83 | 8.55 | 149.89 | 7.11 | 81.79 | 16.79 | 107.40 | 4.48 | 68.22 | 7.34 | 163.69 | 8.55 | 211.69 | 50.56 | 173.23 | 46.45 | 86.33 | 2.02 | 70.82 | 1.61 |
| *HSP42*/YDR171W |  | 51.80 | 4.33 | 262.41 | 24.64 | 104.81 | 25.02 | 109.47 | 8.17 | 108.10 | 15.53 | 68.37 | 2.70 | 470.06 | 123.47 | 193.57 | 55.05 | 79.26 | 2.34 | 48.21 | 2.24 |
| *HSP60*/YLR259C |  | 158.46 | 3.61 | 256.64 | 32.51 | 174.06 | 38.97 | 151.51 | 3.73 | 142.27 | 17.24 | 139.51 | 7.03 | 110.16 | 37.04 | 248.73 | 72.95 | 132.21 | 4.38 | 89.83 | 1.09 |
| *HSP78*/YDR258C |  | 12.90 | 2.75 | 66.99 | 4.82 | 50.28 | 10.04 | 63.94 | 3.13 | 65.23 | 9.26 | 22.51 | 0.72 | 96.77 | 26.65 | 45.67 | 9.65 | 20.02 | 1.08 | 7.33 | 1.07 |
| *HSP82*/YPL240C |  | 104.25 | 3.18 | 470.29 | 50.85 | 160.24 | 35.88 | 134.31 | 5.95 | 150.96 | 12.93 | 62.08 | 6.76 | 208.01 | 48.13 | 209.24 | 33.58 | 79.11 | 3.19 | 35.74 | 2.28 |
| *HSP90*/YMR186W |  | 552.77 | 35.32 | 496.66 | 22.24 | 267.76 | 47.97 | 254.25 | 7.54 | 184.79 | 19.17 | 514.94 | 18.92 | 518.88 | 107.88 | 438.34 | 102.25 | 198.03 | 1.87 | 172.56 | 6.39 |
| *HSP104*/YLL026W |  | 42.61 | 1.73 | 290.27 | 19.66 | 125.23 | 18.90 | 137.27 | 5.35 | 153.39 | 20.57 | 79.28 | 3.68 | 693.60 | 143.89 | 207.07 | 44.15 | 75.12 | 1.11 | 31.43 | 1.41 |
| *HSP150*/YJL159W |  | 447.97 | 5.16 | 297.41 | 33.69 | 598.30 | 141.76 | 525.85 | 20.82 | 529.52 | 66.15 | 311.46 | 16.35 | 315.62 | 76.69 | 303.66 | 83.13 | 217.61 | 5.69 | 113.42 | 5.60 |
| *PGM1*/YKL127W | Glycolysis | 18.72 | 0.51 | 6.53 | 0.66 | 6.87 | 2.46 | 7.12 | 0.68 | 4.37 | 1.69 | 11.65 | 1.75 | 4.59 | 1.14 | 8.07 | 2.60 | 3.89 | 0.19 | 2.09 | 0.40 |
| *PGM2*/YMR105C |  | 5.94 | 0.32 | 51.85 | 13.11 | 38.09 | 8.55 | 55.54 | 1.30 | 32.67 | 9.15 | 14.55 | 1.68 | 20.19 | 12.46 | 34.63 | 11.13 | 12.48 | 0.89 | 6.79 | 0.80 |
| *HXK1*/YFR053C |  | 4.75 | 0.59 | 160.66 | 20.83 | 65.90 | 19.09 | 125.05 | 15.57 | 150.86 | 25.05 | 9.56 | 0.45 | 134.48 | 25.59 | 76.97 | 13.38 | 36.14 | 1.93 | 20.65 | 2.06 |
| *HXK2*/YGL253W |  | 14.33 | 3.00 | 23.09 | 3.70 | 2.68 | 0.87 | 1.50 | 0.76 | 0.85 | 0.14 | 10.18 | 2.00 | 10.80 | 5.97 | 6.07 | 2.79 | 0.93 | 0.64 | 0.39 | 0.16 |
| *GLK1*/YCL040W |  | 8.35 | 0.63 | 96.65 | 5.12 | 64.72 | 13.44 | 56.82 | 6.11 | 43.84 | 4.90 | 24.13 | 2.32 | 59.77 | 10.58 | 151.67 | 45.13 | 54.55 | 1.20 | 19.17 | 1.71 |
| *GAL10*/YBR019C |  | 3.17 | 1.31 | 2.88 | 0.62 | 1.93 | 0.64 | 1.95 | 0.05 | 1.86 | 0.50 | 3.54 | 0.46 | 2.87 | 0.21 | 1.82 | 0.59 | 1.16 | 0.05 | 1.21 | 0.11 |
| *PGI1*/YBR196C |  | 177.20 | 5.34 | 106.38 | 7.59 | 105.85 | 35.55 | 98.05 | 13.88 | 97.45 | 8.92 | 130.64 | 1.30 | 97.39 | 33.90 | 127.14 | 50.40 | 58.29 | 0.95 | 35.67 | 3.23 |
| *FBP1*/YLR377C |  | 1.95 | 0.74 | 1.72 | 0.67 | 1.76 | 0.63 | 1.62 | 0.06 | 2.49 | 0.58 | 3.19 | 0.47 | 2.82 | 0.33 | 2.01 | 0.68 | 1.66 | 0.29 | 1.62 | 0.13 |
| *PFK1*/YGR240C |  | 21.46 | 3.51 | 11.45 | 2.05 | 11.12 | 5.07 | 9.86 | 2.24 | 7.00 | 1.44 | 13.42 | 0.77 | 11.56 | 8.20 | 18.01 | 10.29 | 3.61 | 0.27 | 2.47 | 0.46 |
| *PFK2*/YMR205C |  | 129.50 | 7.61 | 57.63 | 1.56 | 43.95 | 7.87 | 55.17 | 5.09 | 29.99 | 2.66 | 105.53 | 9.40 | 30.98 | 5.88 | 54.04 | 12.15 | 18.04 | 2.15 | 5.91 | 0.45 |
| *FBA1*/YKL060C |  | 741.72 | 36.04 | 592.59 | 87.51 | 492.94 | 79.99 | 549.48 | 54.81 | 443.24 | 83.68 | 597.00 | 57.41 | 532.01 | 180.07 | 599.46 | 177.98 | 252.00 | 14.69 | 191.96 | 19.22 |
| *TPI1*/YDR050C |  | 416.08 | 8.29 | 422.78 | 39.87 | 264.02 | 54.45 | 279.83 | 24.20 | 228.79 | 29.24 | 377.80 | 26.14 | 355.10 | 93.12 | 400.96 | 101.65 | 185.78 | 9.01 | 122.24 | 7.89 |
| *TDH1*/YJL052W |  | 16.37 | 1.81 | 725.99 | 106.24 | 460.92 | 89.11 | 500.17 | 97.16 | 567.11 | 66.51 | 28.14 | 2.08 | 320.45 | 60.49 | 487.23 | 149.09 | 274.84 | 24.74 | 164.80 | 31.70 |
| *TDH2*/YJR009C |  | 982.38 | 76.84 | 1000.76 | 224.48 | 751.47 | 205.62 | 574.93 | 79.89 | 390.60 | 86.09 | 785.43 | 90.12 | 881.76 | 254.80 | 842.08 | 316.90 | 317.18 | 2.98 | 177.56 | 14.05 |
| *TDH3*/YGR192C |  | 1148.03 | 59.12 | 949.64 | 116.73 | 812.08 | 165.68 | 758.61 | 64.13 | 402.54 | 74.41 | 1025.83 | 66.95 | 860.87 | 209.39 | 614.10 | 52.04 | 250.43 | 12.88 | 149.86 | 1.71 |
| *PGK1*/YCR012W |  | 527.75 | 38.19 | 673.51 | 51.07 | 428.96 | 22.65 | 469.66 | 63.09 | 410.43 | 28.53 | 619.54 | 51.22 | 734.77 | 129.28 | 762.32 | 175.47 | 377.98 | 19.33 | 236.43 | 12.57 |
| *GPM1*/YKL152C |  | 517.09 | 17.54 | 702.36 | 108.42 | 579.61 | 177.23 | 453.14 | 67.26 | 435.47 | 50.37 | 512.29 | 56.78 | 619.95 | 187.46 | 729.36 | 303.96 | 313.64 | 18.44 | 276.17 | 42.35 |
| *GPM2*/YDL021W |  | 0.23 | 0.03 | 1.50 | 0.47 | 0.88 | 0.08 | 1.47 | 0.23 | 0.81 | 0.10 | 0.15 | 0.02 | 5.02 | 1.78 | 2.45 | 1.18 | 0.76 | 0.14 | 0.26 | 0.07 |
| *ENO1*/YGR254W |  | 1211.90 | 37.99 | 1393.97 | 146.25 | 1009.15 | 152.59 | 928.94 | 73.94 | 874.66 | 87.24 | 1089.56 | 81.77 | 1097.37 | 264.10 | 1265.84 | 346.48 | 637.59 | 21.76 | 425.17 | 24.61 |
| *ENO2*/YHR174W |  | 917.74 | 44.59 | 874.21 | 104.19 | 532.98 | 116.44 | 455.89 | 61.14 | 226.85 | 24.49 | 771.96 | 39.05 | 724.73 | 205.50 | 879.09 | 235.90 | 394.43 | 6.55 | 324.40 | 50.86 |
| *ERR1*/YOR393W |  | 3.02 | 1.08 | 3.69 | 0.75 | 3.31 | 1.51 | 2.62 | 0.35 | 2.92 | 1.02 | 3.28 | 0.56 | 3.73 | 0.15 | 1.95 | 0.44 | 1.45 | 0.13 | 1.78 | 0.07 |
| *ERR3*/YMR323W |  | 0.82 | 0.31 | 1.01 | 0.32 | 1.06 | 0.48 | 0.73 | 0.03 | 0.63 | 0.16 | 1.11 | 0.23 | 1.19 | 0.21 | 0.66 | 0.23 | 0.38 | 0.03 | 0.47 | 0.07 |
| *CDC19*/YAL038W |  | 691.56 | 45.38 | 567.53 | 80.98 | 412.02 | 107.49 | 372.42 | 60.66 | 311.15 | 41.39 | 589.83 | 60.06 | 649.37 | 186.35 | 720.29 | 284.47 | 293.36 | 15.32 | 261.13 | 53.70 |
| *PYK2*/YOR347C |  | 6.25 | 0.34 | 8.43 | 1.58 | 8.44 | 2.56 | 8.14 | 0.42 | 4.80 | 0.84 | 9.56 | 0.35 | 4.93 | 0.51 | 10.92 | 2.99 | 4.73 | 0.37 | 2.87 | 0.04 |
| *PDA1*/YER178W |  | 54.15 | 3.19 | 23.45 | 4.99 | 34.23 | 10.32 | 34.01 | 5.20 | 25.07 | 2.41 | 28.59 | 7.52 | 20.22 | 6.20 | 48.06 | 19.83 | 16.82 | 0.18 | 7.27 | 1.19 |
| *PDB1*/YBR221C |  | 38.94 | 3.36 | 17.92 | 3.28 | 29.09 | 7.75 | 29.71 | 3.47 | 19.63 | 1.82 | 30.09 | 1.59 | 13.24 | 3.84 | 42.18 | 16.53 | 12.17 | 1.74 | 5.18 | 0.37 |
| *THI3*/YDL080C |  | 10.54 | 0.39 | 5.46 | 0.55 | 6.32 | 1.33 | 9.35 | 0.92 | 5.25 | 0.67 | 10.46 | 0.69 | 7.81 | 1.38 | 5.22 | 1.11 | 4.36 | 0.34 | 4.03 | 0.07 |
| *ARO10*/YDR380W |  | 6.68 | 1.37 | 9.25 | 0.92 | 7.01 | 1.70 | 6.34 | 0.37 | 5.64 | 1.24 | 6.71 | 0.68 | 6.41 | 1.09 | 6.41 | 1.99 | 6.11 | 0.21 | 4.19 | 0.02 |
| *PDC1*/YLR044C |  | 979.83 | 33.36 | 621.77 | 61.32 | 614.10 | 124.99 | 515.49 | 18.86 | 406.47 | 50.58 | 905.93 | 64.36 | 662.44 | 163.54 | 624.71 | 152.80 | 238.48 | 4.01 | 115.71 | 9.34 |
| *PDC5*/YLR134W |  | 739.05 | 65.39 | 458.00 | 47.32 | 458.09 | 82.25 | 374.64 | 17.59 | 300.93 | 43.98 | 637.23 | 55.34 | 504.17 | 128.51 | 467.94 | 143.96 | 162.80 | 8.67 | 73.58 | 7.63 |
| *PDC6*/YGR087C |  | 5.98 | 2.61 | 5.68 | 1.06 | 12.12 | 3.68 | 8.78 | 0.23 | 68.17 | 8.61 | 6.47 | 0.76 | 6.97 | 0.28 | 5.31 | 1.39 | 7.51 | 0.14 | 5.99 | 0.03 |
| *LAT1*/YNL071W |  | 21.45 | 3.22 | 11.55 | 1.14 | 19.11 | 5.48 | 26.10 | 4.02 | 15.43 | 1.54 | 16.41 | 0.37 | 12.30 | 3.51 | 29.59 | 9.53 | 13.29 | 0.46 | 6.26 | 0.54 |
| *ACS1*/YAL054C |  | 0.22 | 0.05 | 0.24 | 0.02 | 0.21 | 0.12 | 0.23 | 0.03 | 0.36 | 0.10 | 0.29 | 0.11 | 1.03 | 0.17 | 0.36 | 0.13 | 0.29 | 0.01 | 0.16 | 0.01 |
| *ACS2*/YLR153C |  | 41.65 | 2.04 | 16.34 | 2.34 | 34.60 | 6.53 | 34.07 | 6.68 | 14.38 | 2.26 | 78.07 | 7.55 | 21.70 | 8.30 | 53.87 | 18.60 | 27.92 | 0.71 | 11.75 | 1.08 |
| *LPD1*/YFL018C |  | 63.88 | 4.28 | 31.08 | 4.01 | 41.74 | 1.42 | 76.65 | 6.36 | 59.02 | 8.24 | 44.08 | 2.98 | 28.88 | 7.85 | 52.01 | 18.93 | 24.39 | 0.94 | 17.98 | 1.31 |
| *IRC15*/YPL017C |  | 0.44 | 0.14 | 0.41 | 0.13 | 0.33 | 0.05 | 0.45 | 0.03 | 0.38 | 0.09 | 0.21 | 0.06 | 0.41 | 0.09 | 0.34 | 0.12 | 0.25 | 0.01 | 0.16 | 0.01 |
| *ALD2*/YMR170C |  | 0.22 | 0.09 | 0.17 | 0.07 | 0.18 | 0.09 | 0.19 | 0.02 | 0.28 | 0.12 | 0.20 | 0.10 | 0.22 | 0.03 | 0.31 | 0.18 | 0.10 | 0.03 | 0.09 | 0.01 |
| *ALD3*/YMR169C |  | 3.05 | 0.83 | 2.32 | 0.45 | 2.81 | 0.81 | 3.01 | 0.05 | 4.43 | 1.01 | 3.57 | 0.67 | 3.61 | 0.21 | 3.07 | 0.86 | 1.83 | 0.09 | 1.54 | 0.03 |
| *ALD4*/YOR374W |  | 9.11 | 0.12 | 53.82 | 7.69 | 78.98 | 21.20 | 71.16 | 1.03 | 61.74 | 7.92 | 10.15 | 1.46 | 114.88 | 15.10 | 53.28 | 15.70 | 28.48 | 1.70 | 14.45 | 0.98 |
| *ALD5*/YER073W |  | 6.75 | 0.24 | 2.96 | 0.53 | 5.70 | 1.93 | 9.05 | 0.27 | 5.96 | 1.35 | 10.12 | 1.82 | 6.53 | 0.67 | 7.03 | 1.82 | 8.99 | 0.36 | 4.14 | 0.05 |
| *ALD6*/YPL061W |  | 250.45 | 33.99 | 53.13 | 2.01 | 54.78 | 22.55 | 19.96 | 3.28 | 13.12 | 2.87 | 133.04 | 12.60 | 34.73 | 16.42 | 14.55 | 2.93 | 7.62 | 0.34 | 8.79 | 1.43 |
| *ADH1*/YOL086C |  | 376.81 | 48.47 | 558.63 | 86.34 | 523.17 | 128.75 | 386.99 | 34.43 | 270.31 | 51.03 | 132.37 | 17.75 | 562.41 | 136.32 | 763.04 | 294.97 | 326.93 | 14.15 | 232.74 | 23.05 |
| *ADH2*/YMR303C |  | 334.27 | 42.51 | 503.53 | 27.25 | 546.79 | 67.03 | 439.95 | 25.40 | 269.52 | 51.33 | 113.93 | 16.59 | 547.96 | 100.91 | 809.69 | 227.41 | 381.52 | 8.67 | 213.80 | 4.56 |
| *ADH3*/YMR083W |  | 46.80 | 4.54 | 17.75 | 1.90 | 59.44 | 9.54 | 61.69 | 1.27 | 54.70 | 6.21 | 23.82 | 1.73 | 14.66 | 2.71 | 94.06 | 28.60 | 41.43 | 2.48 | 22.98 | 1.98 |
| *ADH4*/YGL256W |  | 483.80 | 20.04 | 200.31 | 18.15 | 39.79 | 9.52 | 48.31 | 9.47 | 20.53 | 2.59 | 589.33 | 77.82 | 365.37 | 89.13 | 117.95 | 19.47 | 31.68 | 0.99 | 29.91 | 9.53 |
| *ADH5*/YBR145W |  | 6.00 | 1.37 | 13.67 | 2.47 | 14.13 | 3.37 | 12.96 | 2.39 | 12.32 | 2.63 | 10.51 | 1.61 | 28.78 | 13.76 | 28.85 | 8.22 | 11.71 | 0.32 | 6.68 | 0.77 |
| *ADH6*/YMR318C |  | 45.09 | 1.43 | 14.74 | 1.00 | 16.44 | 3.41 | 15.46 | 0.15 | 11.23 | 2.56 | 44.90 | 2.61 | 15.57 | 2.78 | 24.36 | 6.86 | 12.41 | 0.26 | 7.70 | 0.06 |
| *ADH7* |  | 0.0060 | 0.0010 | 0.0055 | 0.0021 | 0.0049 | 0.0017 | 0.0051 | 0.0010 | 0.0067 | 0.0025 | 0.0021 | 0.0009 | 0.0082 | 0.0008 | 0.0062 | 0.0013 | 0.0029 | 0.0001 | 0.0023 | 0.0012 |
| *SFA1*/YDL168W |  | 68.25 | 4.58 | 101.49 | 12.33 | 118.48 | 24.12 | 141.27 | 4.13 | 138.23 | 16.25 | 59.30 | 7.40 | 109.41 | 22.46 | 138.53 | 48.03 | 61.08 | 2.18 | 34.66 | 4.53 |
| YDR248C | Pentose  Phosphate  pathway | 9.92 | 1.77 | 4.20 | 0.84 | 9.07 | 2.85 | 17.66 | 1.47 | 14.14 | 2.06 | 5.91 | 1.30 | 3.82 | 0.72 | 8.11 | 3.18 | 4.36 | 0.15 | 3.05 | 0.31 |
| *ZWF1*/YNL241C |  | 62.93 | 3.70 | 42.85 | 4.15 | 51.48 | 9.66 | 47.77 | 1.23 | 31.17 | 3.36 | 35.56 | 2.13 | 27.64 | 5.31 | 42.64 | 12.45 | 23.59 | 0.55 | 9.18 | 0.69 |
| *SOL1*/YNR034W |  | 7.85 | 0.35 | 10.44 | 1.75 | 7.02 | 1.55 | 6.67 | 0.17 | 5.74 | 0.92 | 6.00 | 0.82 | 16.36 | 3.03 | 7.49 | 2.41 | 3.98 | 0.10 | 3.10 | 0.19 |
| *SOL2*/YCR073W-A |  | 44.08 | 1.40 | 37.65 | 4.03 | 47.02 | 8.26 | 49.53 | 1.82 | 32.32 | 5.91 | 49.34 | 3.13 | 31.17 | 5.47 | 94.28 | 30.20 | 40.35 | 0.60 | 25.16 | 1.93 |
| *SOL3*/YHR163W |  | 41.79 | 0.82 | 6.28 | 0.91 | 13.75 | 4.00 | 31.13 | 0.69 | 10.09 | 0.95 | 23.91 | 1.18 | 8.72 | 3.12 | 21.26 | 6.37 | 8.75 | 0.52 | 6.03 | 1.65 |
| *SOL4*/YGR248W |  | 0.01 | 0.00 | 0.07 | 0.05 | 0.32 | 0.09 | 0.39 | 0.04 | 0.30 | 0.03 | 0.04 | 0.01 | 0.26 | 0.14 | 0.27 | 0.13 | 0.06 | 0.01 | 0.04 | 0.02 |
| *GND1*/YHR183W |  | 212.19 | 10.42 | 32.57 | 2.17 | 38.25 | 5.00 | 106.01 | 4.92 | 52.41 | 7.15 | 117.45 | 2.26 | 29.38 | 11.01 | 74.08 | 22.28 | 33.84 | 1.17 | 11.55 | 0.96 |
| *GND2*/YGR256W |  | 0.73 | 0.26 | 6.91 | 2.24 | 18.63 | 4.74 | 21.12 | 1.50 | 18.49 | 0.74 | 0.81 | 0.04 | 1.70 | 0.25 | 3.25 | 0.92 | 1.00 | 0.17 | 0.83 | 0.08 |
| *RKI1*/YOR095C |  | 16.35 | 2.35 | 2.43 | 0.26 | 2.77 | 0.68 | 5.58 | 0.16 | 2.07 | 0.62 | 15.16 | 2.74 | 4.15 | 0.72 | 6.17 | 1.92 | 2.94 | 0.06 | 2.23 | 0.27 |
| *RPE1*/YJL121C |  | 29.94 | 3.33 | 5.53 | 1.14 | 10.59 | 1.91 | 14.46 | 1.50 | 9.50 | 1.10 | 21.13 | 2.91 | 6.17 | 2.37 | 16.36 | 4.36 | 7.20 | 0.54 | 6.28 | 0.53 |
| *TKL1*/YPR074C |  | 178.86 | 13.37 | 25.69 | 3.63 | 67.02 | 5.18 | 112.35 | 6.72 | 64.42 | 8.14 | 110.12 | 9.72 | 17.03 | 5.68 | 82.08 | 19.62 | 34.96 | 3.47 | 12.66 | 1.12 |
| *TKL2*/YBR117C |  | 3.28 | 0.91 | 2.93 | 0.63 | 4.81 | 2.10 | 2.59 | 0.46 | 4.08 | 0.63 | 3.65 | 0.54 | 3.51 | 0.23 | 1.84 | 0.51 | 1.66 | 0.03 | 1.75 | 0.01 |
| *RBK1*/YCR036W |  | 3.59 | 0.26 | 1.87 | 0.35 | 3.41 | 0.80 | 3.85 | 0.51 | 5.66 | 0.17 | 3.38 | 0.37 | 1.88 | 0.42 | 4.35 | 1.28 | 3.09 | 0.62 | 2.43 | 0.09 |
| *NQM1*/YGR043C |  | 2.95 | 0.88 | 2.18 | 0.66 | 28.49 | 7.36 | 9.64 | 0.80 | 17.02 | 2.00 | 2.81 | 0.36 | 3.47 | 0.53 | 3.06 | 0.95 | 1.58 | 0.12 | 1.54 | 0.07 |
| *TAL1*/YLR354C |  | 59.77 | 8.43 | 19.92 | 2.06 | 26.26 | 1.94 | 47.22 | 2.92 | 28.66 | 4.15 | 47.22 | 2.99 | 14.38 | 7.95 | 45.73 | 12.94 | 16.85 | 1.34 | 7.72 | 0.43 |
| *PRS1*/YKL181W |  | 44.13 | 7.06 | 6.66 | 0.50 | 10.93 | 2.34 | 19.30 | 1.32 | 17.22 | 2.35 | 20.36 | 1.88 | 5.04 | 2.45 | 21.47 | 9.14 | 8.47 | 0.24 | 5.28 | 0.07 |
| *PRS2*/YER099C |  | 5.48 | 1.02 | 1.65 | 0.42 | 2.83 | 0.77 | 4.62 | 0.32 | 4.32 | 0.76 | 6.39 | 0.41 | 2.51 | 1.10 | 8.43 | 3.91 | 3.90 | 0.13 | 2.62 | 0.22 |
| *PRS3*/YHL011C |  | 19.98 | 3.10 | 3.29 | 0.25 | 4.74 | 1.14 | 8.32 | 0.13 | 7.55 | 0.79 | 16.74 | 0.31 | 4.73 | 1.09 | 11.49 | 3.87 | 6.85 | 0.20 | 3.75 | 0.04 |
| *PRS4*/YBL068W |  | 0.358 | 0.056 | 0.087 | 0.027 | 0.163 | 0.043 | 0.180 | 0.004 | 0.122 | 0.026 | 0.338 | 0.016 | 0.090 | 0.023 | 0.360 | 0.160 | 0.107 | 0.010 | 0.056 | 0.004 |
| *PRS5*/YOL061W |  | 24.58 | 2.37 | 3.96 | 0.33 | 6.97 | 2.05 | 11.07 | 1.22 | 7.99 | 1.55 | 22.78 | 2.74 | 5.59 | 2.52 | 16.81 | 5.65 | 6.04 | 0.46 | 3.23 | 0.08 |
| *PDR1*/YGL013C | PDR  Family | 6.58 | 0.52 | 3.39 | 0.88 | 3.69 | 1.39 | 3.59 | 0.16 | 3.80 | 0.62 | 3.88 | 0.19 | 2.81 | 0.77 | 3.76 | 1.27 | 1.61 | 0.03 | 1.17 | 0.17 |
| *PDR3*/YBL005W |  | 5.46 | 0.32 | 2.97 | 0.64 | 2.77 | 0.83 | 3.77 | 0.04 | 3.00 | 0.63 | 4.48 | 0.30 | 2.85 | 0.38 | 3.52 | 1.15 | 1.74 | 0.07 | 1.06 | 0.17 |
| *PDR5*/YOR153W |  | 56.04 | 4.63 | 6.94 | 0.11 | 5.48 | 0.90 | 4.32 | 0.14 | 4.67 | 1.12 | 12.88 | 0.25 | 2.66 | 0.72 | 7.39 | 0.68 | 3.26 | 0.18 | 1.00 | 0.05 |
| *PDR10*/YOR328W |  | 0.033 | 0.020 | 0.020 | 0.010 | 0.029 | 0.021 | 0.014 | 0.004 | 0.010 | 0.006 | 0.044 | 0.006 | 0.054 | 0.011 | 0.021 | 0.012 | 0.010 | 0.001 | 0.013 | 0.003 |
| *PDR12*/YPL058C |  | 10.75 | 1.40 | 9.14 | 1.60 | 4.91 | 1.95 | 4.68 | 0.25 | 6.06 | 2.10 | 7.11 | 1.09 | 7.05 | 0.97 | 4.32 | 1.73 | 1.80 | 0.21 | 1.66 | 0.08 |
| *PDR15*/YDR406W |  | 9.35 | 0.45 | 11.71 | 2.40 | 10.30 | 2.77 | 15.99 | 1.63 | 11.67 | 3.05 | 6.99 | 1.18 | 7.26 | 1.05 | 6.33 | 1.59 | 2.54 | 0.26 | 1.82 | 0.26 |
| *PDR16*/YNL231C |  | 40.63 | 5.54 | 21.56 | 1.91 | 24.03 | 4.11 | 31.14 | 2.48 | 26.35 | 4.99 | 31.64 | 0.58 | 18.90 | 3.42 | 40.60 | 13.23 | 21.74 | 1.12 | 15.60 | 1.01 |
| *YOR1*/YGR281W |  | 1.95 | 0.08 | 0.70 | 0.17 | 0.71 | 0.29 | 0.42 | 0.20 | 0.32 | 0.09 | 0.90 | 0.17 | 0.51 | 0.26 | 0.80 | 0.43 | 0.11 | 0.03 | 0.11 | 0.05 |
| *SNQ2*/YDR011W |  | 84.97 | 13.41 | 20.67 | 0.47 | 15.85 | 4.36 | 23.69 | 0.88 | 17.12 | 2.54 | 36.45 | 1.19 | 9.77 | 2.30 | 19.67 | 6.10 | 7.23 | 0.42 | 4.19 | 0.48 |
| *RSB1*/YOR049C |  | 18.32 | 4.36 | 18.41 | 3.27 | 18.87 | 1.23 | 18.94 | 0.44 | 18.26 | 1.24 | 19.75 | 0.72 | 19.49 | 0.57 | 20.37 | 1.26 | 19.58 | 0.41 | 19.22 | 0.16 |
| *ICT1*/YLR099C |  | 15.85 | 0.89 | 4.79 | 0.38 | 4.94 | 1.06 | 3.45 | 0.09 | 4.79 | 0.83 | 7.92 | 0.72 | 7.98 | 1.15 | 9.18 | 3.26 | 5.17 | 0.09 | 2.76 | 0.13 |
| *DDI1*/YER143W |  | 22.42 | 0.26 | 22.81 | 3.51 | 26.03 | 6.56 | 21.98 | 0.25 | 31.59 | 4.23 | 13.34 | 0.79 | 14.71 | 1.63 | 26.76 | 8.10 | 13.71 | 0.47 | 8.51 | 0.41 |
| *TPO1*/YLL028W |  | 34.18 | 2.45 | 19.99 | 1.92 | 39.77 | 7.95 | 63.42 | 5.31 | 70.28 | 8.49 | 20.37 | 1.45 | 28.94 | 5.09 | 53.47 | 16.75 | 38.21 | 0.97 | 21.11 | 0.87 |
| *GRE2*/YOL151W |  | 169.89 | 23.89 | 57.60 | 4.03 | 61.94 | 16.87 | 65.98 | 1.57 | 75.31 | 6.30 | 41.24 | 2.23 | 54.29 | 10.75 | 63.16 | 19.32 | 25.98 | 1.76 | 21.26 | 3.30 |
| YMR102C |  | 29.34 | 0.43 | 22.15 | 2.20 | 19.97 | 4.92 | 21.28 | 0.39 | 17.68 | 2.57 | 18.28 | 0.92 | 22.62 | 3.92 | 16.97 | 4.61 | 13.33 | 0.25 | 10.88 | 0.59 |
| YLL056C |  | 3.29 | 0.72 | 3.49 | 1.48 | 2.88 | 0.73 | 3.52 | 0.08 | 7.57 | 0.99 | 3.40 | 0.18 | 4.51 | 1.66 | 3.63 | 1.08 | 3.29 | 0.32 | 2.82 | 0.10 |
| YLR346C |  | 1.19 | 0.34 | 1.19 | 0.41 | 1.02 | 0.45 | 0.64 | 0.05 | 0.86 | 0.16 | 2.00 | 0.14 | 4.66 | 1.06 | 1.01 | 0.37 | 1.26 | 0.15 | 1.48 | 0.20 |
| *MSN2*/ YMR037C | Transcription factors | 31.87 | 0.41 | 24.05 | 2.78 | 20.30 | 3.58 | 25.04 | 0.80 | 15.83 | 2.00 | 30.79 | 2.32 | 37.54 | 5.35 | 20.23 | 5.12 | 12.39 | 0.38 | 7.02 | 0.22 |
| *MSN4*/ YKL062W |  | 7.23 | 2.09 | 5.68 | 0.87 | 8.75 | 1.49 | 17.33 | 1.86 | 21.83 | 3.05 | 6.87 | 0.85 | 6.52 | 0.09 | 4.53 | 0.90 | 3.22 | 0.12 | 2.50 | 0.16 |
| *YAP1*/ YML007W |  | 20.09 | 0.88 | 11.86 | 2.34 | 10.28 | 2.25 | 12.58 | 0.39 | 9.68 | 1.06 | 13.15 | 1.22 | 22.23 | 4.69 | 13.23 | 4.49 | 6.99 | 0.08 | 3.56 | 0.39 |
| *HSF1*/ YGL073W |  | 9.53 | 0.96 | 8.56 | 1.91 | 7.62 | 1.80 | 9.66 | 0.31 | 8.30 | 1.07 | 6.54 | 0.90 | 10.39 | 2.81 | 7.41 | 2.67 | 4.36 | 0.26 | 2.41 | 0.21 |
