# Supplementary material for: Quantitative transcription dynamic analysis reveals candidate genes and key regulators for ethanol tolerance in Saccharomyces cerevisiae
Source: BMC Microbiol. 2010 Jun 10;10:169. doi: 10.1186/1471-2180-10-169 (PMC2903563; doi:10.1186/1471-2180-10-169)
Supplement: Additional file 3 — Gene Ontology (GO) categories and terms of candidate and key genes for ethanol tolerance and fermentation under stress in Saccharomyces cerevisiae. [file 1471-2180-10-169-S3.DOC]

Additional File 3. Gene Ontology (GO) categories and terms of candidate and key genes for ethanol tolerance and fermentation under stress in *Saccharomyces cerevisiae*

| GO ID | GO term | Gene(s) |
| --- | --- | --- |
| Cellular component | | |
| GO:0005737 | Cytoplasm | *NTH2, ETR1, HSP26, TKL2, TPS1, PGI1, GLK1, GPM2, GPD1, SFA1, NTH1, SNQ2, HSP42, YDR248C, HSP78, PRO1, PDA1, HSP12, LPD1, GSY1, HXK1, ERG26, ERG4, TRP5, HSF1, GUP1, TDH3, PFK1, SOL4, GND2, HTD2, SOL3, GND1, TDH1, ERG20, TDH2, UGP1, FBA1, MSN4, PGM1, PRS1, HSP104, TPO1, PUT1, GSY2, YAP1, DAK1, TSL1, MSN2, ADH3, PGM2, TPS3, ADH2, ZWF1, ADH1, GRE2, GCY1, PDR5, PYK2, ALD4, IRC15, ALD6, HSP82, ATH1, TKL1, GPH1* |
| GO:0005739 | Mitochondrion | *NTH2, ETR1, PGI1, SFA1, SNQ2, HSP78, PDA1, LPD1, GSY1, HXK1, HSF1, TDH3, PFK1, HTD2, GND1, TDH1, TDH2, FBA1, PUT1, ADH3, PDR5, PYK2, ALD4, ALD6* |
| GO:0005634 | Nucleus | *HSP26, TKL2, GPD1, HSP12, PDR1, TRP5, HSF1, NQM1, SOL4, SOL3, MSN4, HSP104, YAP1, MSN2, GRE2, GCY1* |
| GO:0016020 | Membrane | *HSP30, SNQ2, PDR15, DDI1, HSP12, ERG26, GUP1, YOR1, ELO1, TPO1, PDR5, PDR12, GUP2* |
| GO:0005624 | Membrane fraction | *PGI1, GLK1, HSP30, SNQ2, HSP12, TDH3, GND2, YOR1, TDH1, TDH2, UGP1, ADH1* |
| GO:0005886 | Plasma membrane | *HSP30, SNQ2, DDI1, HSP12, GUP1, YOR1, TPO1, PDR5, PDR12* |
| GO:0005618 | Cell wall | *TDH3, TDH1, HSP150, TDH2, ATH1* |
| GO:0005783 | Endoplasmic reticulum | *ERG26, ERG4, GUP1, ERG20* |
| GO:0005856 | Cytoskeleton | *HSP42, IRC15* |
| GO:0005773 | Vacuole | *TPO1, ATH1* |
| GO:0005777 | Peroxisome | *GPD1* |
| GO:0005933 | Cellular bud | *TPO1* |
| GO:0012505 | Endomembrane system | *ERG26* |
| GO:0005576 | Extracellular region | *HSP150* |
| GO:0005575 | Cellular component unknown | *ICT1, YMR102C, ERR1, HSP32* |
| Other | Other | *ADH7*, *HSP31* |
| Biological process | | |
| GO:0044262 | Cellular carbohydrate metabolic process | *NTH2, TKL2, TPS1, PGI1, GLK1, NTH1, YDR248C, GSY1, HXK1, GUP1, TDH3, PFK1, SOL4, GND2, SOL3, GND1, TDH1, TDH2, UGP1, FBA1, PGM1, PRS1, HSP104, GSY2, DAK1, TSL1, PGM2, TPS3, ZWF1, ADH1, GCY1, ATH1, TKL1, GPH1* |
| GO:0006950 | Response to stress | *HSP26, TPS1, HSP30, GPD1, NTH1, SNQ2, HSP78, HSP12, HSF1, GND1, MSN4, HSP104, YAP1, DAK1, TSL1, MSN2, TPS3, ZWF1, GCY1, ALD6, HSP82, ATH1* |
| GO:0006091 | Generation of precursor metabolites and energy | *ETR1, PGI1, GLK1, GSY1, HXK1, TDH3, PFK1, TDH1, TDH2, UGP1, FBA1, PGM1, GSY2, ADH3, PGM2, ADH2, ADH1, GPH1* |
| GO:0042221 | Response to chemical stimulus | *ETR1, PGI1, GLK1, GSY1, HXK1, TDH3, PFK1, TDH1, TDH2, UGP1, FBA1, PGM1, GSY2, ADH3, PGM2, ADH2, ADH1, GPH1* |
| GO:0006766 | Vitamin metabolic process | *TKL2, PGI1, GPD1, SOL4, GND2, SOL3, GND1, ADH3, ADH2, ZWF1, ADH1, ALD4, ALD6, TKL1* |
| GO:0051186 | Cofactor metabolic process | *TKL2, PGI1, GPD1, SOL4, GND2, SOL3, GND1, ADH3 ,ADH2, ZWF1, ADH1, ALD4, ALD6, TKL1* |
| GO:0006810 | Transport | *GLK1, HSP78, PDR15, DDI1, HXK1, GUP1, YOR1, TPO1, PDR5, PDR12, GUP2, HSP82* |
| GO:0044255 | Cellular lipid metabolic process | *ETR1, ERG26, ERG4, GUP1, HTD2, ERG20, ELO1, ICT1, GRE2* |
| GO:0006519 | Cellular amino acid and derivative metabolic process | *SFA1, PRO1, LPD1, TRP5, PUT1, ADH3, ADH2, ADH1* |
| GO:0016070 | RNA metabolic process | *PDR1, HSF1, MSN4, YAP1, MSN2* |
| GO:0006350 | Transcription | *PDR1, HSF1, MSN4, YAP1, MSN2* |
| GO:0046483 | Heterocycle metabolic process | *SFA1, PRO1, TRP5, PRS1, PUT1* |
| GO:0006457 | Protein folding | *HSP26, HSP78, HSP104, HSP82* |
| GO:0007010 | Cytoskeleton organization | *HSP42, HSF1, IRC15* |
| GO:0051276 | Chromosome organization | *IRC15, HSP82* |
| GO:0007005 | Mitochondrion organization | *HSP78, HSP82* |
| GO:0007049 | Cell cycle | *HSF1, IRC15* |
| GO:0006464 | Protein modification process | *GUP1, UGP1* |
| GO:0031505 | Fungal-type cell wall organization | *HSP150, PRS1* |
| GO:0019725 | Cellular homeostasis | *GPD1, PGM2* |
| GO:0006259 | DNA metabolic process | *IRC15, HSP82* |
| GO:0045333 | Cellular respiration | *ETR1* |
| GO:0006412 | Translation | *HSP78* |
| GO:0070271 | Protein complex biogenesis | *HSP82* |
| GO:0007059 | Chromosome segregation | *IRC15* |
| GO:0007165 | Signal transduction | *HSF1* |
| GO:0007126 | Meiosis | *IRC15* |
| GO:0006725 | Cellular aromatic compound metabolic process | *TRP5* |
| GO:0044257 | Cellular protein catabolic process | *DDI1* |
| GO:0016192 | Vesicle-mediated transport | *DDI1* |
| GO:0008150 | Biological process unknown | *GPM2, HSP31, NQM1, YMR102C, ERR1, HSP32* |
| Other | Other | *ADH7, PDA1, PYK2* |
| Molecular function | | |
| GO:0016740 | Transferase activity | *TKL2, TPS1, GLK1, YDR248C, PRO1, GSY1, HXK1, GUP1, NQM1, PFK1, ERG20, ELO1, UGP1, PRS1, ICT1, GSY2, DAK1, TSL1, TPS3, PYK2, IRC15, GUP2, TKL1, GPH1* |
| GO:0016491 | Oxidoreductase activity | *ETR1, ADH7, GPD1, SFA1, PDA1, LPD1, ERG26, ERG4, TDH3, GND2, GND1, TDH1, TDH2, PUT1, ADH3, ADH2, ZWF1, ADH1, GRE2, GCY1, ALD4, ALD6* |
| GO:0016787 | Hydrolase activity | *NTH2, NTH1, SNQ2, HSP78, PDR15, HSP31, SOL4, YOR1, SOL3, HSP104, TSL1, TPS3, PDR5, PDR12, HSP82, HSP32, ATH1* |
| GO:0005515 | Protein binding | *HSP26, HSP42, HSP78, HSP31, DDI1, HSP104, IRC15, HSP82, HSP32* |
| GO:0005215 | Transporter activity | *SNQ2, PDR15, YOR1, TPO1, PDR5, PDR12* |
| GO:0030528 | Transcription regulator activity | *PDR1, HSF1, MSN4, YAP1, MSN2* |
| GO:0003677 | DNA binding | *PDR1, HSF1, MSN4, YAP1, MSN2* |
| GO:0016853 | Isomerase activity | *PGI1, GPM2, PGM1, PGM2* |
| GO:0016829 | Lyase activity | *TRP5, HTD2, FBA1, ERR1* |
| GO:0008233 | Peptidase activity | *HSP31*, *HSP32* |
| GO:0030234 | Enzyme regulator activity | *TSL1, TPS3* |
| GO:0016779 | Nucleotidyltransferase activity | *UGP1* |
| GO:0005198 | Structural molecule activity | *HSP150* |
| GO:0003674 | Molecular function unknown | *HSP30, GPM2, HSP12, YMR102C* |
