# Supplementary material for: Quantitative transcription dynamic analysis reveals candidate genes and key regulators for ethanol tolerance in Saccharomyces cerevisiae
Source: BMC Microbiol. 2010 Jun 10;10:169. doi: 10.1186/1471-2180-10-169 (PMC2903563; doi:10.1186/1471-2180-10-169)
Supplement: Additional file 4 — Primers used for mRNA expression analysis by real-time qRT-PCR using SYBR Green. [file 1471-2180-10-169-S4.DOC]

Additional File 4. Primers used for mRNA expression analysis by real-time qRT-PCR using SYBR Green

| ID | Sequence 5’-3’ | Direction | Amplicon (bp) | Gene / ORF |
| --- | --- | --- | --- | --- |
| B2M_L | agcgtcctccaaagattcaa | Forward |  |  |
| B2M_R | tccccattcttcagcaaatc | Reverse | 127 | *B2M* |
| MSG_L | gatgagcacagccttgtgaa | Forward |  |  |
| MSG_R | cctccacgttcttggtgagt | Reverse | 112 | *MSG* |
| CAB_L | agacagcactcccatggttc | Forward |  |  |
| CAB_ R | aatcccatcagtgccatctc | Reverse | 109 | *CAB* |
| RBS1_L | gcttggaattcgagttggag | Forward |  |  |
| RBS1_R | gagaagcatcagtgcaacca | Reverse | 123 | *RBS1* |
| ACTB_L | gctctcttccagccttcctt | Forward |  |  |
| ACTB_R | tagaggtccttgcggatgtc | Reverse | 104 | *ACTB* |
| ACC1_L | aagggtcattgtaccgcttg | Forward |  |  |
| ACC1_R | cggagaagtaaccccaaaca | Reverse | 124 | *ACC1*/YNR016C |
| FAS1_L | tatgctgctttggcctcttt | Forward |  |  |
| FAS1_R | gttggatctgcccaactcat | Reverse | 120 | *FAS1*/YKL182W |
| FAS2_L | agattgccgatgaacctgtc | Forward |  |  |
| FAS2_R | ccaccgaccaagtctttgat | Reverse | 118 | *FAS2*/YPL231W |
| ELO1_L | gtatcgccatgggctgtatt | Forward |  |  |
| ELO1_R | gacagtgtctgcgaactcca | Reverse | 115 | *ELO1*/YJL196C |
| FEN1_L | ggttcaacaactgccacctt | Forward |  |  |
| FEN1_R | ctggttttggtgcctttacg | Reverse | 107 | *FEN1*/YCR034W |
| SUR4_L | atccagctgagcaattcgag | Forward |  |  |
| SUR4_R | gatagcttggccaccaaaga | Reverse | 113 | *SUR4*/YLR372W |
| OLE1_L | cgacaagaagggaaacgaaa | Forward |  |  |
| OLE1_R | tggttgttcggagatgtgaa | Reverse | 106 | *OLE1*/YGL055W |
| IFA38_L | cacactccattcctgttcca | Forward |  |  |
| IFA38_R | ccacaatctttggtgcaatg | Reverse | 117 | *IFA38*/YBR159W |
| PHS1_L | gcgtcaccattgtccttctt | Forward |  |  |
| PHS1_R | ggctgtccaacttttgggta | Reverse | 104 | *PHS1*/YJL097W |
| TSC13_L | tggtcttttcgtgctttcag | Forward |  |  |
| TSC13_R | ttcaatgggacacggatctt | Reverse | 111 | *TSC13*/YDL015C |
| HFA1_L | ctgggtcgcctatgtttgtt | Forward |  |  |
| HFA1_R | ccgcctttgtatggaacagt | Reverse | 125 | *HFA1*/YMR207C |
| MCT1a-L | ggaactcacacgttgatgga | Forward |  |  |
| MCT1a-R | ttgcacagtcctgcttgaac | Reverse | 120 | *MCT1*/YOR221C |
| CEM1_L | ccgcataccttaaacctgga | Forward |  |  |
| CEM1_R | cttcggattagctcccacaa | Reverse | 102 | *CEM1*/YER061C |

| OAR1_L | ccgcccttgaaacagaaata | Forward |  |  |
| --- | --- | --- | --- | --- |
| OAR1_R | agcctgcgcagttaatcaat | Reverse | 103 | *OAR1*/YKL055C |
| HTD2a_L | ttcctgttcttcccaccatc | Forward |  |  |
| HTD2a_R | agctctccttgtccccaaat | Reverse | 128 | *HTD2*/YHR067W |
| ETR1_L | accgagttatcccattgcag | Forward |  |  |
| ETR1_R | cagatacagttgccgcagaa | Reverse | 120 | *ETR1*/YBR026C |
| ERG10_L | gtcgtagctggtggttgtga | Forward |  |  |
| ERG10_R | cccatctctttcgacaccat | Reverse | 114 | *ERG10*/YPL028W |
| ERG13_L | catctgtttatgccgccttt | Forward |  |  |
| ERG13_R | cagctaaaccggaaccgtaa | Reverse | 102 | *ERG13*/YML126C |
| HMG1_L | ccatcaactggatcgaaggt | Forward |  |  |
| HMG1_R | aactcaaccaatgcggaaac | Reverse | 109 | *HMG1*/YML075C |
| HMG2_L | ggtgcctgcaagatatggtt | Forward |  |  |
| HMG2_R | aaagcaaatcgcctgctaga | Reverse | 124 | *HMG2*/YLR450W |
| ERG12_L | tggactgcttgtctcaatcg | Forward |  |  |
| ERG12_R | agcaccggtaagttttgtgg | Reverse | 100 | *ERG12*/YMR208W |
| ERG8_L | acagctttggcctccttttt | Forward |  |  |
| ERG8_R | cgcttccaattttaccctga | Reverse | 118 | *ERG8*/YMR220W |
| MVD1_L | caggtccaaatgctgtgttg | Forward |  |  |
| MVD1_R | ttcttgtcccatccaggaac | Reverse | 100 | *MVD1*/YNR043W |
| ERG20_L | tgcacctgaagacaaagtcg | Forward |  |  |
| ERG20_R | caatgcgacaggcaagtaga | Reverse | 106 | *ERG20*/YJL167W |
| ERG9_L | tgaaagcatgggtcttttcc | Forward |  |  |
| ERG9_R | tgaggagcgtattgtgacca | Reverse | 114 | *ERG9*/YHR190W |
| ERG1_L | agaaaggaattgcacccaga | Forward |  |  |
| ERG1_R | taacgtgaccgtgcatagga | Reverse | 103 | *ERG1*/YGR175C |
| ERG7_L | cacctgttaaccaggcgttt | Forward |  |  |
| ERG7_R | ataccctgtggaccatggaa | Reverse | 121 | *ERG7*/YHR072W |
| ERG11_L | tttcggtggtggtagacaca | Forward |  |  |
| ERG11_R | tggaacggtcttaccctctg | Reverse | 121 | *ERG11*/YHR007C |
| ERG24_L | tggtttcatgctagcgtttg | Forward |  |  |
| ERG24_R | accgacaactttcacccatc | Reverse | 112 | *ERG24*/YNL280C |
| ERG25_L | caacgctaccctttcaggtc | Forward |  |  |
| ERG25_R | ggcccagtatttctccatga | Reverse | 106 | *ERG25*/YGR060W |
| ERG26_L | cgtactgtgtggaaggcaga | Forward |  |  |
| ERG26_R | tggctctttacccagcatct | Reverse | 120 | *ERG26*/YGL001C |
| ERG27_L | ggtcaaggaggttttcacca | Forward |  |  |
| ERG27_R | ggaaaataagccccatgtca | Reverse | 104 | *ERG27*/YLR100W |

| ERG6_L | ccgataaagatgccgaagaa | Forward |  |  |
| --- | --- | --- | --- | --- |
| ERG6_R | atggaaagaggaaccccaac | Reverse | 110 | *ERG6*/YML008C |
| ERG2_L | gggtcttcaacaatgctggt | Forward |  |  |
| ERG2_R | tgtgtgcccttcagtaccaa | Reverse | 110 | *ERG2*/YMR202W |
| ERG3_L | gtctaccgtgctctgcacaa | Forward |  |  |
| ERG3_R | tgtggtacgagatggattgc | Reverse | 109 | *ERG3*/YLR056W |
| ERG5_L | ttggttacagcagcattgga | Forward |  |  |
| ERG5_R | ccatttgagcacagttttcg | Reverse | 121 | *ERG5*/YMR015C |
| ERG4_L | acggctatgtgacaccacaa | Forward |  |  |
| ERG4_R | gtaagccatgtcccaggttg | Reverse | 110 | *ERG4*/YGL012W |
| ERG28_L | tggttgccctattccacttc | Forward |  |  |
| ERG28_R | ccaaaccaaagaggtggttg | Reverse | 107 | *ERG28*/YER044C |
| ERG29_L | ttgcgtgtattgaggctgag | Forward |  |  |
| ERG29_R | tgacaccgttggaaatcgta | Reverse | 118 | *ERG29*/YMR134W |
| PRO1_L | cgtgtccagtggtggtattg | Forward |  |  |
| PRO1_R | cccatctcccgatcaatcta | Reverse | 122 | *PRO1*/YDR300C |
| PRO2_L | cgtggttccaatgccttagt | Forward |  |  |
| PRO2_R | caaatctgcgtcttcatcca | Reverse | 108 | *PRO2*/YOR323C |
| PRO3_L | cgaaagcacttacggtcaca | Forward |  |  |
| PRO3_R | gacgctcttcacaccattca | Reverse | 109 | *PRO3*/YER023W |
| PUT1_L | caacggcaatagtgtgatgg | Forward |  |  |
| PUT1_R | aagctgttcaaggtcgcaat | Reverse | 111 | *PUT1*/YLR142W |
| PUT2_L | acatccgcaagtccaatttc | Forward |  |  |
| PUT2_R | atttccaactcggggtcttt | Reverse | 125 | *PUT2*/YHR037W |
| NTH1_L | aagagctttccaagcaagca | Forward |  |  |
| NTH1_R | ccgagaccgttaggatggta | Reverse | 105 | *NTH1*/YDR001C |
| NTH2_L | gccaccattcctaaccgata | Forward |  |  |
| NTH2_R | gctcgacatccacacctctt | Reverse | 136 | *NTH2*/YBR001C |
| ATH1_L | ctacgggaccttgtgtcgat | Forward |  |  |
| ATH1_R | ctcgtcatcaatcccttcgt | Reverse | 101 | *ATH1*/YPR026W |
| TPS2_L | cagcagtcctactgccaaca | Forward |  |  |
| TPS2_R | gtaatgctggacgggagaaa | Reverse | 124 | *TPS2*/YDR074W |
| TPS1_L | aactttcaagggctgcaaga | Forward |  |  |
| TPS1_R | ctccattctggatgctcgtt | Reverse | 114 | *TPS1*/YBR126C |
| TPS3_L | tgggagtccaaccaagaaac | Forward |  |  |
| TPS3_R | ggacgttggaggttctgaaa | Reverse | 120 | *TPS3*/YMR261C |
| TSL1_L | ctgtgaggttcaccccagtt | Forward |  |  |
| TSL1_R | ccttgccatctttcacgaat | Reverse | 106 | *TSL1*/YML100W |

| UGP1_L | ccacatgatcgagactggtg | Forward |  |  |
| --- | --- | --- | --- | --- |
| UGP1_R | ttgggcgacttccaataaac | Reverse | 121 | *UGP1*/YKL035W |
| GPH1_L | atctggccacccatgaatta | Forward |  |  |
| GPH1_R | tgctaaagaagccgacgttt | Reverse | 102 | *GPH1*/YPR160W |
| GSY1_L | tgatgtggaccaagaagctg | Forward |  |  |
| GSY1_R | gcagtgatttgcgacacagt | Reverse | 108 | *GSY1/YFR015C* |
| GSY2_L | ccagatgggattttgcctaa | Forward |  |  |
| GSY2_R | caaccatgaaaatggcctct | Reverse | 125 | *GSY2*/YLR258W |
| TRP2_L | gagaaaggcgtggggtttat | Forward |  |  |
| TRP2_R | tgcaagtaagcaatgccatc | Reverse | 118 | *TRP2*/YER090W |
| TRP3a_L | cgaagggacccatttgttta | Forward |  |  |
| TRP3a_R | tgtaacgaaccgtgaaacca | Reverse | 118 | *TRP3*/YKL211C |
| TRP4_L | tctggcatattgatccgaca | Forward |  |  |
| TRP4_R | ctttagggccgtatgaagca | Reverse | 117 | *TRP4*/YDR354W |
| TRP1_L | gggacaggtgaacttttgga | Forward |  |  |
| TRP1_R | cgcatcaccaacattttctg | Reverse | 120 | *TRP1*/YDR007W |
| TRP5_L | tattggccgtccttcttcac | Forward |  |  |
| TRP5_R | tgtgagatcccgtgtggtta | Reverse | 107 | *TRP5*/YGL026C |
| GPD1_L | acccgacaatttggttgcta | Forward |  |  |
| GPD1_R | gctacagatacggggcaaaa | Reverse | 103 | *GPD1*/YDL022W |
| GPD2_L | tcaaaggctgggtttaggtg | Forward |  |  |
| GPD2_R | ctgagcaggtggtgatcaga | Reverse | 119 | *GPD2*/YOL059W |
| HOR2_L | ggtgcagttaagctgtgcaa | Forward |  |  |
| HOR2_R | ccagatgctcgaaccatttt | Reverse | 106 | *HOR2*/YER062C |
| RHR2_L | aagttcgctccagactttgc | Forward |  |  |
| RHR2_R | caacttgacagcacctggaa | Reverse | 108 | *RHR2/*YIL053W |
| GUT1_L | accgattgcatctacggttc | Forward |  |  |
| GUT1_R | attatggtggctctggcatc | Reverse | 105 | *GUT1*/YHL032C |
| GUT2_L | gggaacgtcgtccaaatcta | Forward |  |  |
| GUT2_R | tgtttacgctcgttgagtgc | Reverse | 123 | *GUT2*/YIL155C |
| GCY1_L | aaaggcattgtggttgaagc | Forward |  |  |
| GCY1_R | tgtccgggttgaacgttatt | Reverse | 110 | *GCY1*/YOR120W |
| DAK1a_L | gcgtctggcgttttattgat | Forward |  |  |
| DAK1a_R | tctctgccaactgcaacatc | Reverse | 137 | *DAK1*/YML070W |
| PFS1_L | gacgacaacacgaacagtgg | Forward |  |  |
| PFS1_R | cgtaccgtggggatacaaat | Reverse | 113 | *PFS1*/YHR185C |
| GUP1_L | gattccgtggaggctgttta | Forward |  |  |
| GUP1_R | atgccaagctctccagaatg | Reverse | 118 | *GUP1*/YGL084C |

| GUP2_L | cccttgcaacaggctatgat | Forward |  |  |
| --- | --- | --- | --- | --- |
| GUP2_R | caggtgcatcaataccatcg | Reverse | 115 | *GUP2*/YPL189W |
| HSP10_L | gacagcatccgggttgtatt | Forward |  |  |
| HSP10_R | tcagcgtccctgaaaagaat | Reverse | 210 | *HSP10*/YOR020C |
| HSP12_L | cgcaggtagaaaaggattcg | Forward |  |  |
| HSP12_R | tcggccttgtcagtgatgta | Reverse | 105 | *HSP12*/YFL014W |
| HSP26_L | cttgtccctgttcccatctg | Forward |  |  |
| HSP26_R | gacaccaggaaccacgactt | Reverse | 109 | *HSP26*/YBR072W |
| HSP30_L | ttggactggtgttcaagctg | Forward |  |  |
| HSP30_R | caggacaagaaccaggcaat | Reverse | 117 | *HSP30*/YCR021C |
| HSP31_L | aaaagaggtgaatgccgatg | Forward |  |  |
| HSP31_R | atttcggaagcaatgtcctg | Reverse | 108 | *HSP31*/YDR533C |
| HSP32_L | gagctttattggtggcgaag | Forward |  |  |
| HSP32_R | atagtcgctggcgttgactt | Reverse | 109 | *HSP32*/YPL280W |
| HSP40_L | caagaattgtcccagccatt | Forward |  |  |
| HSP40_R | cggaatcatagtttgcacca | Reverse | 120 | *HSP40*/YNL064C |
| HSP42_L | taccagttccctggacaagc | Forward |  |  |
| HSP42_R | gtcttgtagtgccgctgtca | Reverse | 115 | *HSP42*/YDR171W |
| HSP60_L | acaagaccagccaagcagat | Forward |  |  |
| HSP60_R | tcgtaacccttggcaaaatc | Reverse | 104 | *HSP60*/YLR259C |
| HSP78_L | gatggatcccaatcagcaac | Forward |  |  |
| HSP78_R | gctcttgcgatttcttcgtc | Reverse | 123 | *HSP78*/YDR258C |
| HSP82_L | actccaaagccagagcaaaa | Forward |  |  |
| HSP82_R | gagcttccatgaaggctttg | Reverse | 124 | *HSP82*/YPL240C |
| HSP90_L | ccgctttgttgacttctggt | Forward |  |  |
| HSP90_R | tggagcggtttctgtttctt | Reverse | 119 | *HSP90*/YMR186W |
| HSP104_L | tgaagtcgctgaaccaagtg | Forward |  |  |
| HSP104_R | tggctaattgagcagcagtg | Reverse | 125 | *HSP104*/YLL026W |
| HSP150_L | tctgagccttggtccacttt | Forward |  |  |
| HSP150_R | atgatgcgctggatgtagtg | Reverse | 115 | *HSP150*/YJL159W |
| PGM1_L | ccataggcgtaaccctgaaa | Forward |  |  |
| PGM1_R | tgctcgcattcgatatgttc | Reverse | 117 | *PGM1*/YKL127W |
| PGM2_L | tgctttgttcgacgctaaaa | Forward |  |  |
| PGM2_R | gttctccggatgatgcttgt | Reverse | 148 | *PGM2*/YMR105C |
| HXK1_L | tgctgtcgacgaacaatctc | Forward |  |  |
| HXK1_R | caacatcaagcccttctcgt | Reverse | 124 | *HXK1*/YFR053C |
| HXK2_L | ccaattccattgggtttcac | Forward |  |  |
| HXK2_R | gcaacattggaacaacatcg | Reverse | 130 | *HXK2*/YGL253W |

| GLK1_L | ctgacgacctgtttgggttt | Forward |  |  |
| --- | --- | --- | --- | --- |
| GLK1_R | agtgaaccccagtttcatgg | Reverse | 113 | *GLK1*/YCL040W |
| GAL10_L | acttgggttccggtaaaggt | Forward |  |  |
| GAL10_R | tgctcttctgcccgtaactt | Reverse | 101 | *GAL10*/YBR019C |
| PGI1_L | ctgaaggtgccatttggaat | Forward |  |  |
| PGI1_R | agcatcgtgggtagaaatgg | Reverse | 122 | *PGI1*/YBR196C |
| FBP1_L | ccatggttgctgatgttcac | Forward |  |  |
| FBP1_R | cattgggaaggcctcataaa | Reverse | 113 | *FBP1*/YLR377C |
| PFK1_L | ccaactgttgagcacgaaga | Forward |  |  |
| PFK1_R | ttggtttcgttttcccaaag | Reverse | 101 | *PFK1*/YGR240C |
| PFK2_L | tcgtggtggtccagaataca | Forward |  |  |
| PFK2_ | taccttcgcgcttcttgaat | Reverse | 119 | *PFK2*/YMR205C |
| FBA1_L | tgctttcggtaactgtcacg | Forward |  |  |
| FBA1_R | caatggcttttcttccttgc | Reverse | 118 | *FBA1*/YKL060C |
| TPI1_L | cttgaaggcttctggtgctt | Forward |  |  |
| TPI1_R | cgaacttggtcttgtcagca | Reverse | 146 | *TPI1*/YDR050C |
| TDH1_L | ctcacgcttccatcttcgat | Forward |  |  |
| TDH1_R | aagccttggcaacatattcg | Reverse | 135 | *TDH1*/YJL052W |
| TDH2_L | cgtcgaagttgttgctttga | Forward |  |  |
| TDH2_R | tgtcatcgtgggaaacttca | Reverse | 113 | *TDH2*/YJR009C |
| TDH3_L | gttgctttgaacgacccatt | Forward |  |  |
| TDH3_L | gcttgtcatcgtgggaaact | Reverse | 106 | *TDH3*/YGR192C |
| PGK1_L | cttgccagtcgacttcatca | Forward |  |  |
| PGK1_R | cctttgcaacagtagcagca | Reverse | 149 | *PGK1*/YCR012W |
| GPM1_L | cgacgcttcttctccattct | Forward |  |  |
| GPM1_R | tgtcaatgaccaaagccaaa | Reverse | 101 | *GPM1*/YKL152C |
| GPM2_L | aagcacaccatccaatgtca | Forward |  |  |
| GPM2_R | ttcgattagctcggcagaat | Reverse | 148 | *GPM2*/YDL021W |
| ENO1_L | aaggtggtgttgctccaaac | Forward |  |  |
| ENO1_R | gcacagtccaaaccgatctt | Reverse | 112 | *ENO1*/YGR254W |
| ENO2_L | gctgacttggttgtcggttt | Forward |  |  |
| ENO2_R | acagccttgtcacccaattc | Reverse | 122 | *ENO2*/YHR174W |
| ERR1a_L | agtccagcccagttagcaga | Forward |  |  |
| ERR1a_R | atcttcggcgtagggatctt | Reverse | 84 | *ERR1*/YOR393W |
| ERR3_L | aaggacgggaaatacgacct | Forward |  |  |
| ERR3_R | tcttccagggaaatgattgg | Reverse | 128 | *ERR3*/YMR323W |
| CDC19_L | tgatgatgttgaagcccgta | Forward |  |  |
| CDC19_R | ccttgaaaccttggatggaa | Reverse | 101 | *CDC19*/YAL038W |

| PYK2_L | aattgaaatcctggcacctg | Forward |  |  |
| --- | --- | --- | --- | --- |
| PYK2_R | tgaatccagcatctgagtcg | Reverse | 106 | *PYK2*/YOR347C |
| PDA1_L | gttgcctgaatcttccttcg | Forward |  |  |
| PDA1_R | ttgtacaaggcgtcacaagc | Reverse | 141 | *PDA1*/YER178W |
| PDB1_L | ccagactgccaacatcattg | Forward |  |  |
| PDB1_R | tctgacggtcatcgtctttg | Reverse | 119 | *PDB1*/YBR221C |
| THI3_L | atgttccacaggctctgctt | Forward |  |  |
| THI3_R | ggaattctggagttggcgta | Reverse | 111 | *THI3*/YDL080C |
| ARO10_L | caagaccactcaaacgctca | Forward |  |  |
| ARO10_R | tgcacttcagaatggtgctc | Reverse | 130 | *ARO10*/YDR380W |
| PDC1_L | acaccatcttggctttggtc | Forward |  |  |
| PDC1_ | cgaaagctgggaattgagtc | Reverse | 126 | *PDC1*/YLR044C |
| PDC5_L | cacgttgttggtgttccatc | Forward |  |  |
| PDC5_R | tcagtgatcatggcagtggt | Reverse | 134 | *PDC5*/YLR134W |
| PDC6_L | ggagattgaccccaacaaga | Forward |  |  |
| PDC6_R | atacggctttaacccccatc | Reverse | 106 | *PDC6*/YGR087C |
| LAT1a_L | gaacgctacagcaaacgaca | Forward |  |  |
| LAT1a_R | aaccagtaggcattggcatc | Reverse | 105 | *LAT1*/YNL071W |
| ACS1_L | tgatgacgcgctaagagaga | Forward |  |  |
| ACS1_R | ctgttgcccaatccaaatct | Reverse | 110 | *ACS1*/YAL054C |
| ACS2_L | ctaccgtgccattctttggt | Forward |  |  |
| ACS2_R | attgatggccatggtgattt | Reverse | 115 | *ACS2*/YLR153C |
| LPD1_L | actggtcacggtcatgtcaa | Forward |  |  |
| LPD1_R | tagtcaatgccggcttcttt | Reverse | 113 | *LPD1*/YFL018C |
| IRC15_L | acgatgcaaatgaattgctg | Forward |  |  |
| IRC15_R | aactggacggcttgcttaaa | Reverse | 127 | *IRC15*/YPL017C |
| ALD2b_L | ttgttggaaattgcaaggtg | Forward |  |  |
| ALD2b_R | tgacaacaccaggtggaaaa | Reverse | 131 | *ALD2*/YMR170C |
| ALD3_L | ccagggctgcttttgataac | Forward |  |  |
| ALD3_R | tctctaatgcggcaagtgtg | Reverse | 117 | *ALD3*/YMR169C |
| ALD4_L | aggccattacaaaccatcca | Forward |  |  |
| ALD4_R | gatttaccacccagctccaa | Reverse | 127 | *ALD4*/YOR374W |
| ALD5_L | cgaacagccaacagggttat | Forward |  |  |
| ALD5_R | caacggcttcatcaacatca | Reverse | 143 | *ALD5*/YER073W |
| ALD6_L | tgagcacaggtttgaaggtg | Forward |  |  |
| ALD6_R | ttgcttaacaccaccgaatg | Reverse | 104 | *ALD6*/YPL061W |
| ADH1_L | tacgccggtatcaaatggtt | Forward |  |  |
| ADH1_R | tcgtgggtgtaaccagacaa | Reverse | 107 | *ADH1*/YOL086C |

| ADH2_L | actggccattgccaactaag | Forward |  |  |
| --- | --- | --- | --- | --- |
| ADH2_R | accatttgataccggcgtag | Reverse | 120 | *ADH2*/YMR303C |
| ADH3_L | aatggctgaacggttcttgt | Forward |  |  |
| ADH3_R | gaattttggcggcttgaata | Reverse | 147 | *ADH3*/YMR083W |
| ADH4_L | tgtcacagctggtttgaagg | Forward |  |  |
| ADH4_R | cgatttccccaccgttagta | Reverse | 125 | *ADH4*/YGL256W |
| ADH5b_L | tggatggtactggcttcaca | Forward |  |  |
| ADH5b_R | tcagcaagattgacgtttgg | Reverse | 100 | *ADH5*/YBR145W |
| ADH6_L | aagtcggtcaacgtgttggt | Forward |  |  |
| ADH6_R | agccgtcttcataaggctga | Reverse | 129 | *ADH6*/YMR318C |
| ADH7_L | atttccaacgcaaaggattg | Forward |  |  |
| ADH7_R | agatccgcagataccacagg | Reverse | 111 | *ADH7*/YCR105W |
| SFA1_L | atgtgcaaaaaggcgatacc | Forward |  |  |
| SFA1_R | tggcaccaaattgagaacaa | Reverse | 150 | *SFA1*/YDL168W |
| YDR248C_L | cgttgggactggttgaaaaa | Forward |  |  |
| YDR248C_R | ggcatgtgtgcctaatcaaa | Reverse | 130 | YDR248C |
| ZWF1_L | ccagtttttgaatgcctcgt | Forward |  |  |
| ZWF1_R | acagatggttctgcatcacg | Reverse | 140 | *ZWF1*/YNL241C |
| SOL1_L | aaaggcgcctgtaatcaaga | Forward |  |  |
| SOL1_R | gcatcgtcgtctacaaacca | Reverse | 111 | *SOL1*/YNR034W |
| SOL2_L | ccgatgttcgacttgttcct | Forward |  |  |
| SOL2_R | accacccatgcaagtttctc | Reverse | 104 | *SOL2*/YCR073W-A |
| SOL3_L | cttgctgaacgaaacaacca | Forward |  |  |
| SOL3_L | ctttggcgtctttcaacaca | Reverse | 104 | *SOL3*/YHR163W |
| SOL4_L | tcgttaggggagctgctaaa | Forward |  |  |
| SOL4_R | ttggttccgaccatttcatt | Reverse | 100 | *SOL4*/YGR248W |
| GND1_L | tgtctgctgatttcggtttg | Forward |  |  |
| GND1_R | atggtcgaccttggattgag | Reverse | 119 | *GND1*/YHR183W |
| GND2_L | ggtaagccattggtggaaaa | Forward |  |  |
| GND2_R | ctgacaaacaacgagcgaaa | Reverse | 136 | *GND2*/YGR256W |
| RKI1_L | ctttggaggatgccaagaga | Forward |  |  |
| RKI1_R | ttcggcaacataaaccactg | Reverse | 116 | *RKI1*/YOR095C |
| RPE1_L | tggaagacatgatgccaaaa | Forward |  |  |
| RPE1_R | tggtaccagcgacaataacg | Reverse | 138 | *RPE1*/YJL121C |
| TKL1_L | agcctacggttgggaagttt | Forward |  |  |
| TKL1_R | gcaaggaaccgtaaccaatg | Reverse | 143 | *TKL1*/YPR074C |
| TKL2_L | gtccaacgcaccaacctatt | Forward |  |  |
| TKL2_R | tggtgttcgaccagatttga | Reverse | 131 | *TKL2*/YBR117C |

| RBK1_L | gatactttcctgggcggttt | Forward |  |  |
| --- | --- | --- | --- | --- |
| RBK1_R | tacagtggcatgctttcagc | Reverse | 131 | *RBK1*/YCR036W |
| NQM1_L | attggctgcttcaaagttgg | Forward |  |  |
| NQM1_R | tatccatggcgttctcgatt | Reverse | 110 | *NQM1*/YGR043C |
| TAL1_L | acactggtgatttcggctct | Forward |  |  |
| TAL1_R | acttggcgtaagttggttgc | Reverse | 102 | *TAL1*/YLR354C |
| PRS1_L | tcagacaaggtgaccatcca | Forward |  |  |
| PRS1_L | tgcaattcgctgtcagattc | Reverse | 148 | *PRS1*/YKL181W |
| PRS2_L | aatggatcacggtgctaagg | Forward |  |  |
| PRS2_R | tcgacaatgtccaaatccaa | Reverse | 147 | *PRS2*/YER099C |
| PRS3_L | catgcttcccaaattcaagg | Forward |  |  |
| PRS3_R | ccagcatctggcgaaattat | Reverse | 128 | *PRS3*/YHL011C |
| PRS4_L | ccaaattgcatctgcaagaa | Forward |  |  |
| PRS4_R | gcaaccaactttgcggtaat | Reverse | 108 | *PRS4*/YBL068W |
| PRS5_L | tgctcccataatttccaagc | Forward |  |  |
| PRS5_R | tcttcaacgagctctcagca | Reverse | 113 | *PRS5*/YOL061W |
| PDR1a_L | aatccggatctcccaagtct | Forward |  |  |
| PDR1a_R | ttgtttctcactggggaagg | Reverse | 110 | *PDR1*/YGL013C |
| PDR3_L | atactgccgaacggagaaga | Forward |  |  |
| PDR3_R | ctgaaatccttcggcaagag | Reverse | 130 | *PDR3*/YBL005W |
| PDR5_L | tgacgcttttgcatcagttc | Forward |  |  |
| PDR5_R | gagaaaaccgcgacaatgtt | Reverse | 150 | *PDR5*/YOR153W |
| PDR10_L | ctcgggtttgaatcaaggaa | Forward |  |  |
| PDR10_R | aaaagggaagattggctcgt | Reverse | 135 | *PDR10*/YOR328W |
| PDR12_L | ttggccgaaattggtaactc | Forward |  |  |
| PDR12_R | cgcagacaaatggtacatgg | Reverse | 81 | *PDR12*/YPL058C |
| PDR15_L | ggttggtccatttggatttg | Forward |  |  |
| PDR15_R | ttgggcacaagggaatctac | Reverse | 99 | *PDR15*/YDR406W |
| PDR16_L | tcccacctattggtgtaggg | Forward |  |  |
| PDR16_R | acgggtcagtgggtcaataa | Reverse | 149 | *PDR16*/YNL231C |
| YOR1b_L | ttccctgcaattttggctat | Forward |  |  |
| YOR1b_R | atgaaaaacccaccgaaaaa | Reverse | 98 | *YOR1*/YGR281W |
| SNQ2_L | tatcaaaagctggccaatcc | Forward |  |  |
| SNQ2_R | gtttgtccacccttcctcaa | Reverse | 103 | *SNQ2*/YDR011W |
| RSB1_L | catcgctacggtcactttga | Forward |  |  |
| RSB1_R | gtgattggaatcgaggtgct | Reverse | 75 | *RSB1*/YOR049C |
| ICT1_L | actactgcagacgccaaggt | Forward |  |  |
| ICT1_R | tgtggaaatgccactggtta | Reverse | 139 | *ICT1*/YLR099C |

| DDI1_L | atacaggggctcaaacaacg | Forward |  |  |
| --- | --- | --- | --- | --- |
| DDI1_R | attttgacttgggcttggtg | Reverse | 148 | *DDI1*/YER143W |
| TPO1_L | tcggtatgatggtgtgtgct | Forward |  |  |
| TPO1_R | ggatagtagcccgtccaaca | Reverse | 169 | *TPO1*/YLL028W |
| GRE2_L | cgtatcggaggccagattta | Forward |  |  |
| GRE2_R | atgggtagcaccagaacctg | Reverse | 112 | *GRE2*/YOL151W |
| YMR102C_L | acaccaagggaaagcatgtc | Forward |  |  |
| YMR102C_R | agcaccagaaaaagcctcaa | Reverse | 129 | YMR102C |
| YLL056C_L | actggtgcttctggcttcat | Forward |  |  |
| YLL056C_R | gaaggatttttgcagcagga | Reverse | 136 | YLL056C |
| YLR346C_L | tgtcccatcgggttagtttc | Forward |  |  |
| YLR346C_R | atctgcctgaaccaacaacg | Reverse | 106 | YLR346C |
| MSN2_L | aatttgggcacatccacttc | Forward |  |  |
| MSN2_R | ggtgagctgcttgtgttgaa | Reverse | 135 | YMR037C |
| MSN4_L | tcgtcacgcaaacaagaaac | Forward |  |  |
| MSN4_R | attccggcgaagatatgcta | Reverse | 143 | YKL062W |
| YAP1a_L | agccaattgacacacccaat | Forward |  |  |
| YAP1a_R | ttgtcgttgtcgttgtcgtt | Reverse | 100 | YML007W |
| HSF1_L | aacatccctctggacaatgc | Forward |  |  |
| HSF1_R | tctccaagattcgcttcgtt | Reverse | 134 | YGL073W |
